# Efficacy of tofacitinib on enthesitis in patients with active psoriatic arthritis: analysis of pooled data from two phase 3 studies

**DOI:** 10.1186/s13075-023-03108-5

**Published:** 2023-08-22

**Authors:** Philip J. Mease, Ana-Maria Orbai, Oliver FitzGerald, Mohamed Bedaiwi, Dona L. Fleishaker, Rajiv Mundayat, Pamela Young, Philip S. Helliwell

**Affiliations:** 1 Rheumatology Research, Swedish Medical Center/Providence St. Joseph Health and University of Washington School of Medicine, Seattle Rheumatology Associates, 601 Broadway, Suite 600, Seattle, WA 98122 USA; 2 Division of Rheumatology, Johns Hopkins University School of Medicine, Baltimore, MD USA; 3 Conway Institute for Biomolecular Research, University College Dublin, Dublin, Ireland; 4 Division of Rheumatology, College of Medicine, King Saud University, Riyadh, Saudi Arabia; 5 Pfizer Inc, Groton, CT USA; 6 Pfizer Inc, New York, NY USA; 7 Pfizer Inc, Collegeville, PA USA; 8 Leeds Institute of Rheumatic and Musculoskeletal Medicine, University of Leeds, Leeds, UK

**Keywords:** Enthesitis, Patient-reported outcomes, Psoriatic arthritis, Spondyloarthritis, Tofacitinib

## Abstract

**Background:**

Tofacitinib is an oral Janus kinase inhibitor for the treatment of psoriatic arthritis (PsA). This post hoc analysis assessed tofacitinib efficacy on enthesitis by baseline location and severity, and impact on disease activity and patient-reported outcomes (PROs), in patients with PsA.

**Methods:**

Data were pooled from two phase 3 studies (NCT01877668/NCT01882439) in patients with PsA receiving tofacitinib 5 or 10 mg twice daily to month (M)6 or placebo to M3. Endpoints were: change from baseline in Leeds Enthesitis Index (LEI) or Spondyloarthritis Research Consortium of Canada Enthesitis Index (SPARCC); proportions of patients with enthesitis, relapsed enthesitis after resolution, de novo enthesitis*,* low disease activity (LDA) or remission (minimal disease activity/very low disease activity; Psoriatic Arthritis Disease Activity Score; Disease Activity Index for Psoriatic Arthritis, and Composite Psoriatic Disease Activity in Psoriatic Arthritis); and PROs (Functional Assessment of Chronic Illness Therapy-Fatigue [FACIT-F] total and arthritis pain Visual Analog Scale scores). Descriptive statistics were generated by visit and treatment. Change from baseline in PROs was evaluated by multivariate linear regression.

**Results:**

Seven hundred ten patients from two studies were included: 479 had LEI > 0; 545 had SPARCC > 0; and 136 had LEI = 0 and SPARCC = 0 at baseline. At baseline, among patients with LEI > 0 or SPARCC > 0, mean LEI and SPARCC across treatments and enthesitis locations/severities ranged from 1.0–4.4 and 1.3–9.4, respectively. Across several baseline enthesitis locations/severities, changes from baseline in LEI and SPARCC up to M3 were greater with tofacitinib (-2.0–0.4 and -3.5–0.2) vs placebo (-‍0.9–‍0.4 and -1.5–1.1). Enthesitis at M6 was more common in patients with greater baseline enthesitis severity. At M6, ≤ 40% of patients with baseline LEI > 0 or SPARCC > 0 whose enthesitis had resolved by M1/M3 experienced a relapse, and < 14% of patients with baseline LEI = 0 and SPARCC = 0 had de novo enthesitis. LDA/remission rates generally increased with tofacitinib over time. Baseline LEI location was significantly associated with change from baseline in arthritis pain score, while baseline SPARCC severity was significantly associated with change from baseline in FACIT-F total and arthritis pain scores.

**Conclusion:**

Tofacitinib treatment resulted in improvements in enthesitis in patients with PsA, regardless of baseline location or severity.

**Trial registration:**

NCT01877668;NCT01882439.

**Supplementary Information:**

The online version contains supplementary material available at 10.1186/s13075-023-03108-5.

## Background

Psoriatic arthritis (PsA) is a chronic inflammatory disease with musculoskeletal and dermatologic manifestations [1]. Enthesitis, defined as inflammation where the tendon, ligament, or joint capsule insert in the bone, has been reported in 35–50% of patients with PsA [2]. Enthesitis has been associated with greater PsA disease activity [3, 4]. Moreover, enthesitis severity has been associated with radiographic peripheral and axial joint damage [5]. Patients with vs without enthesitis generally reported worse functional status, greater fatigue and pain, and reduced work productivity [3, 4]. When deciding on treatment strategies for PsA, enthesitis has been recognized as an important domain to be considered [6].

Tofacitinib is an oral Janus kinase inhibitor for the treatment of PsA. Efficacy and safety of tofacitinib 5 mg twice daily (BID; recommended dosage) [7, 8] and 10 mg BID have been demonstrated in two phase 3 studies of patients with active PsA [9, 10], and in an open-label, long-term extension study [11]. In the two phase 3 studies, tofacitinib treatment was associated with greater improvements in enthesitis vs placebo by month 3 [9, 10].

In this post hoc analysis, the effects of tofacitinib on enthesitis, its impact on PsA disease activity, and patient-reported outcomes (PROs) in patients with vs without enthesitis at baseline, were further evaluated. Development of de novo enthesitis was also investigated.

## Patients and methods

### Study design

This post hoc analysis included pooled data from two phase 3 studies of tofacitinib for the treatment of active PsA: OPAL Broaden (NCT01877668) [9] and OPAL Beyond (NCT01882439) [10]. Details of these trials have been reported previously [9, 10].

OPAL Broaden was a 12-month study of tofacitinib in tumor necrosis factor inhibitor (TNFi)-naïve patients with an inadequate response to a conventional synthetic disease-modifying antirheumatic drug (csDMARD). Patients received tofacitinib 5 or 10 mg BID, adalimumab 40 mg subcutaneous injection once every 2 weeks, or placebo (advancing to tofacitinib 5 or 10 mg BID at month 3) [9]. OPAL Beyond was a 6-month study of tofacitinib in patients with an inadequate response to TNFi. Patients received tofacitinib 5 or 10 mg BID or placebo (advancing to tofacitinib 5 or 10 mg BID at month 3) [10]. In both studies, patients also received a stable background dose of a single csDMARD.

This post hoc analysis included patients receiving tofacitinib 5 or 10 mg BID to month 6, or placebo to month 3.

Both studies were conducted in accordance with Good Clinical Practice and the Declaration of Helsinki. The study protocols were reviewed and approved by the Institutional Review Boards and/or an Independent Ethics Committee at each study center, and all patients provided written, informed consent.

### Assessment

**Fig. 1:**
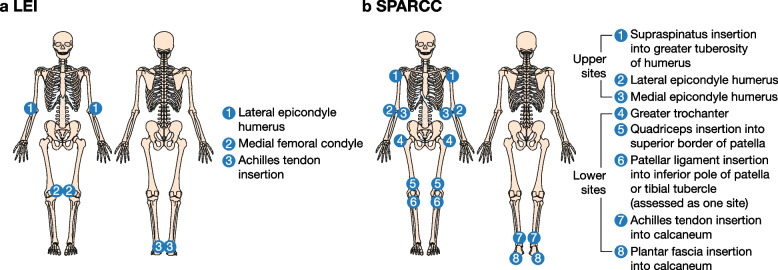
Enthesitis sites evaluated by LEI or SPARCC. LEI, Leeds Enthesitis Index; SPARCC, Spondyloarthritis Research Consortium of Canada Enthesitis Index. Adapted from Mease PJ, et al. J Rheumatol. 2017;44:599–608. Reproduced with permission

In OPAL Broaden and Beyond, presence of enthesitis in patients was determined at baseline, and at months 1, 3, and 6, by a blinded, qualified assessor using the Leeds Enthesitis Index (LEI) [12] and Spondyloarthritis Research Consortium of Canada Enthesitis Index (SPARCC) [13]. To generate LEI scores, three bilateral enthesitis sites were assessed for tenderness: lateral epicondyle humerus, medial femoral condyle, and Achilles tendon insertion (Fig. 1a). To generate SPARCC scores, eight bilateral enthesitis sites were assessed for tenderness: supraspinatus insertion into greater tuberosity of humerus, lateral epicondyle humerus, medial epicondyle humerus, greater trochanter, quadriceps insertion into superior border of patella, patellar ligament insertion into inferior pole of patella or tibial tubercle (assessed as one site), Achilles tendon insertion into calcaneum, and plantar fascia insertion into calcaneum (Fig. 1b). A score was assigned dichotomously for each site assessed by LEI and SPARCC, where 0 = no tenderness and 1 = tenderness. LEI (range 0–6) and SPARCC (range 0–16) scores were calculated as the sum of the site scores, with higher scores indicating greater severity.

Patients were categorized based on the presence (LEI > 0 or SPARCC > 0) or absence (LEI = 0 and SPARCC = 0) of enthesitis at baseline. Patients with enthesitis were further stratified by enthesitis location and severity at baseline. Enthesitis location was determined as the individual sites assessed for LEI (Fig. 1a) and the location of the sites assessed for SPARCC (upper sites, lower sites, or both upper and lower sites; Fig. 1b). Enthesitis severity was determined by the number of affected sites, with a higher number indicating greater severity: 1, 2, or 3–6 affected sites for LEI and 1–2, > 2– ≤ 5, or > 5 affected sites for SPARCC.

Data at baseline and months 1, 3, and 6, from OPAL Broaden and Beyond, were included in this analysis. Individual patient-level data across the two studies were pooled, and data-handling conventions that were utilized in each study, including the handling of missing data, were also applied across the pooled data.

The following endpoints were assessed in patients with LEI > 0 or SPARCC > 0 at baseline: change from baseline in LEI or SPARCC, and the proportions of patients with enthesitis or with relapsed enthesitis (assessed at months 3 and 6 only) after resolution at months 1 or 3. The development of de novo enthesitis in patients with LEI = 0 and SPARCC = 0 at baseline was also assessed.

Disease activity measures assessed were: the proportion of patients achieving low disease activity (LDA) and remission, based on minimal disease activity (MDA; ≥ 5/7 criteria)/very low disease activity (7/7 MDA criteria), Psoriatic Arthritis Disease Activity Score (PASDAS; > 1.9– < 3.2/ ≤ 1.9 [near remission]), Disease Activity Index for Psoriatic Arthritis (DAPSA; > 4– ≤ 14/ ≤ 4), and Composite Psoriatic Disease Activity in Psoriatic Arthritis (CPDAI; > 2– ≤ 4/ ≤ 2). Detailed descriptions of the disease activity measures are presented in Supplementary Table 1 (see Additional file 1).

PROs included Functional Assessment of Chronic Illness Therapy-Fatigue (FACIT-F) total score (range 0–52; higher scores indicate less fatigue) [14] and arthritis pain (assessed by Visual Analog Scale [VAS]; range 0–100 mm) [15].

### Statistical analyses

Demographics and baseline disease characteristics were reported for patients who received ≥ 1 dose of study treatment. Descriptive statistics were generated for each endpoint by visit and treatment arm. Binary endpoints were analyzed using Cochran-Mantel–Haenszel statistics, with non-responder imputation for missing values. The 95% confidence intervals (CIs) were derived based on normal approximation. When compared with placebo, tofacitinib responses were defined as “greater,” “lower,” or “higher” if the corresponding 95% CIs did not overlap.

Multivariate linear regression analyses based on backward selection criteria were conducted to determine the effects of baseline enthesitis location and severity on change from baseline in FACIT-F total score and arthritis pain (VAS) at months 3 and 6. Baseline covariates included were: the respective PRO being assessed; enthesitis in LEI locations (lateral epicondyle humerus [yes/no], medial femoral condyle [yes/no], Achilles tendon insertion [yes/no]); enthesitis severity based on LEI (0 vs 1, 2, or 3–6 affected sites); enthesitis in SPARCC locations (upper sites only [yes/no], lower sites only [yes/no], both upper and lower sites [yes/no]); and enthesitis severity based on SPARCC (0 vs 1–2, > 2– ≤ 5, or > 5 affected sites). In the pooled tofacitinib analysis, a variable for dose group (tofacitinib 5 mg BID vs 10 mg BID) was included in the model as a covariate. Statistical significance was defined as *p* value < 0.05. No adjustments were made for multiple comparisons.

## Results

### Demographics and baseline disease characteristics

In total, 710 patients were analyzed: 479 (67.5%) had LEI > 0; 545 (76.8%) had SPARCC > 0; and 136 (19.2%) had LEI = 0 and SPARCC = 0 at baseline. Demographics and baseline disease characteristics were similar across treatments and baseline location or severity, with some exceptions (Tables 1 and 2, and Supplementary Table 2). Overall, patients with LEI > 0 or patients with SPARCC > 0 had higher mean baseline C-reactive protein (CRP) (8.3–19.7 mg/L) and arthritis pain (VAS) scores (44.2–62.8), compared with patients with LEI = 0 and SPARCC = 0 (7.1–9.8 mg/L and 45.9–51.0, respectively). Mean PASDAS, DAPSA, and CPDAI were also higher in patients with LEI > 0 or patients with SPARCC > 0 (5.6–7.0, 33.6–66.1, and 8.9–12.4, respectively) vs patients with LEI = 0 and SPARCC = 0 (5.1–5.3, 29.2–33.2, and 7.3–8.0, respectively).

**Table 1:** Demographics and baseline disease characteristics of patients with LEI > 0 at baseline

	**Patients with LEI > 0 (*N* = 479)**					
	**Location**^**a**^			**Severity**^**b**^		
	**Lateral epicondyle humerus** ***N* = 331**	**Medial femoral condyle** ***N* = 285**	**Achilles tendon insertion** ***N* = 260**	**1 site** ***N* = 105**	**2 sites** ***N* = 127**	**3–6 sites** ***N* = 246**
**Tofacitinib 5 mg BID**	***N* = 102**	***N* = 94**	***N* = 87**	***N* = 37**	***N* = 47**	***N* = 73**
**Tofacitinib 10 mg BID**	***N* = 119**	***N* = 100**	***N* = 92**	***N* = 31**	***N* = 37**	***N* = 95**
***Placebo***	***N* = *110***	***N* = *91***	***N* = *81***	***N* = *37***	***N* = *43***	***N* = *78***
Female, *n* (%)	58 (56.9)	49 (52.1)	47 (54.0)	19 (51.4)	26 (55.3)	39 (53.4)
	80 (67.2)	66 (66.0)	55 (59.8)	14 (45.2)	20 (54.1)	66 (69.5)
	*68 (61.8)*	*60 (65.9)*	*50 (61.7)*	*15 (40.5)*	*26 (60.5)*	*53 (67.9)*
Age, years, mean (SD)	50.8 (11.8)	49.2 (11.6)	48.7 (12.2)	50.0 (11.6)	49.4 (11.5)	49.7 (12.1)
	50.6 (11.6)	50.2 (11.5)	50.5 (11.6)	48.2 (13.5)	48.4 (11.5)	51.4 (11.3)
	*49.2 (13.0)*	*49.3 (12.3)*	*49.6 (12.4)*	*48.1 (13.2)*	*50.0 (13.0)*	*49.6 (12.7)*
Race, White, *n* (%)	96 (94.1)	89 (94.7)	83 (95.4)	35 (94.6)	46 (97.9)	69 (94.5)
	112 (94.1)	94 (94.0)	88 (95.7)	28 (90.3)	34 (91.9)	92 (96.8)
	*104 (94.5)*	*85 (93.4)*	*77 (95.1)*	*34 (91.9)*	*41 (95.3)*	*73 (93.6)*
BMI, kg/m^2^, mean (SD)	30.2 (6.4)	31.0 (6.9)	30.8 (6.3)	29.1 (7.3)	30.7 (7.1)	30.4 (6.0)
	30.7 (6.3)	31.0 (6.5)	30.6 (6.8)	29.3 (5.6)	29.3 (6.9)	31.1 (6.5)
	*29.7 (5.8)*	*29.5 (5.8)*	*30.4 (6.1)*	*29.9 (4.7)*	*29.3 (5.9)*	*30.0 (6.2)*
PsA duration, years, mean (SD)	7.6 (6.6)	9.6 (8.5)	8.7 (7.7)	10.4 (9.4)	7.5 (7.1)	8.5 (7.2)
	6.9 (5.9)	7.5 (5.8)	7.6 (6.3)	7.1 (5.2)	6.8 (6.9)	7.5 (5.9)
	*7.5 (6.0)*	*8.5 (7.8)*	*8.0 (6.8)*	*10.0 (8.9)*	*9.5 (7.1)*	*6.8 (5.9)*
CRP, mg/L, mean (SD)	9.8 (16.1)	11.3 (18.0)	13.8 (22.5)	17.4 (26.9)	17.1 (29.4)	8.3 (8.3)
	11.0 (20.9)	16.3 (29.9)	13.6 (27.8)	14.7 (21.1)	8.9 (15.1)	14.5 (28.7)
	*10.6 (21.0)*	*11.2 (18.9)*	*10.5 (17.7)*	*17.7 (33.4)*	*9.7 (11.3)*	*9.4 (16.5)*
LEI, mean (SD)	3.3 (1.6)	3.4 (1.6)	3.3 (1.7)	1.0 (0.0)	2.0 (0.0)	4.2 (1.1)
	3.7 (1.7)	4.0 (1.6)	3.9 (1.8)	1.0 (0.0)	2.0 (0.0)	4.4 (1.1)
	*3.3 (1.6)*	*3.5 (1.5)*	*3.4 (1.7)*	*1.0 (0.0)*	*2.0 (0.0)*	*4.1 (1.1)*
SPARCC, mean (SD) [*N1*]^c^	7.2 (3.8) [95]	7.2 (3.9) [88]	6.9 (4.2) [82]	2.7 (1.8) [33]	5.0 (2.4) [44]	8.4 (3.6) [70]
	7.6 (4.3) [116]	8.3 (4.1) [95]	8.1 (4.4) [89]	2.3 (1.7) [30]	5.2 (2.3) [35]	8.9 (3.7) [92]
	*6.6 (3.8) [105]*	*7.0 (3.6) [85]*	*6.7 (3.7) [76]*	*2.8 (1.8) [36]*	*4.5 (2.7) [39]*	*8.0 (3.4) [74]*
Dactylitis presence, DSS > 0, *n* (%)	56 (54.9)	56 (59.6)	53 (60.9)	19 (51.4)	25 (53.2)	46 (63.0)
	65 (54.6)	53 (53.0)	52 (56.5)	15 (48.4)	22 (59.5)	51 (53.7)
	*59 (53.6)*	*50 (54.9)*	*48 (59.3)*	*17 (45.9)*	*21 (48.8)*	*46 (59.0)*
TJC68, mean (SD) [*N1*]^d^	26.2 (14.4) [49]	24.8 (15.1) [41]	21.8 (14.3) [34]	18.2 (11.6) [18]	21.2 (10.5) [28]	28.3 (16.0) [28]
	25.8 (13.4) [50]	24.9 (11.8) [36]	27.4 (14.5) [33]	17.4 (9.5) [13]	22.6 (16.5) [16]	28.3 (12.3) [35]
	*25.7 (16.3) [43]*	*29.1 (16.5) [42]*	*26.5 (15.4) [32]*	*13.9 (8.0) *[12]	*25.3 (14.5) *[21]	*29.6 (16.4) [32]*
SJC66, mean (SD) [*N1*]^d^	15.5 (12.7) [49]	12.7 (8.6) [41]	15.6 (12.6) [34]	11.0 (7.2) [18]	14.6 (11.2) [28]	16.1 (12.6) [28]
	12.4 (8.0) [50]	13.5 (8.5) [36]	14.8 (9.6) [33]	10.3 (5.9) [13]	11.4 (7.4) [16]	14.2 (8.7) [35]
	*13.2 (10.8) [43]*	*13.9 (11.0) [42]*	*11.9 (10.3) [32]*	*7.9 (5.1) *[12]	*13.0 (8.5) *[21]	*14.1 (11.6) [32]*
PASDAS, mean (SD) [*N1*]^d^	6.4 (1.1) [98]	6.5 (1.1) [90]	6.6 (1.1) [83]	5.9 (1.3) [27]	6.4 (1.1) [38]	6.8 (1.1) [52]
	6.5 (1.1) [116]	6.7 (1.1) [97]	6.7 (1.1) [90]	6.4 (1.0) [22]	6.3 (1.2) [29]	7.0 (1.1) [65]
	*6.3 (1.1) [108]*	*6.4 (1.2) [89]*	*6.5 (1.1) [79]*	*5.8 (1.2) *[24]	*6.8 (0.9) *[29]	*6.7 (1.0) [58]*
DAPSA, mean (SD) [*N1*]^d^	54.8 (25.1) [101]	51.5 (24.3) [93]	52.6 (24.1) [86]	41.9 (23.3) [27]	49.3 (18.5) [38]	60.1 (26.2) [54]
	56.8 (26.9)	57.0 (26.2)	60.5 (26.8)	44.1 (21.2) [22]	49.3 (25.8) [29]	63.9 (27.5) [66]
	*50.3 (26.0)*	*52.8 (25.8)*	*53.1 (25.0)*	*33.6 (15.6) *[24]	*50.6 (21.3) *[29]	*59.9 (26.5) [58]*
CPDAI, mean (SD) [*N1*]^e^	11.0 (2.2) [62]	11.3 (2.2) [61]	11.4 (2.1) [60]	11.0 (2.1) [20]	10.7 (1.9) [27]	11.9 (2.2) [41]
	11.3 (2.5) [70]	11.7 (2.2) [57]	11.9 (2.1) [52]	11.1 (2.5) [14]	10.9 (2.3) [24]	12.4 (2.2) [44]
	*10.8 (2.6) [67]*	*11.1 (2.2) [61]*	*11.2 (2.4) [55]*	*10.3 (2.6) *[18]	*12.1 (1.7) *[25]	*11.5 (2.4) [41]*
FACIT-F total score, mean (SD) [*N1*]^d^	26.3 (11.0) [101]	26.2 (11.0) [93]	25.0 (12.1) [86]	25.4 (12.3)	25.0 (10.4)	26.0 (11.6) [72]
	25.0 (10.5)	24.9 (9.8)	24.3 (9.1)	27.2 (10.8)	30.1 (10.6)	23.1 (8.9)
	*27.5 (11.0)*	*26.2 (10.3)*	*27.6 (10.3)*	*31.2 (9.5)*	*26.6 (12.3)*	*26.7 (9.9)*
Arthritis pain (VAS), mean (SD) [*N1*]^d^	58.0 (21.2) [101]	57.6 (21.0) [93]	60.5 (21.0) [86]	55.1 (30.1)	60.9 (18.0)	58.1 (19.7) [72]
	59.3 (21.4)	61.0 (22.1)	59.8 (22.6)	61.4 (22.5)	50.6 (24.8)	62.0 (20.6)
	*53.7 (23.8)*	*57.3 (23.2)*	*56.5 (22.6)*	*48.2 (25.4)*	*59.4 (21.4)*	*55.7 (22.9)*

^a^Each site was assessed bilaterally, and results were combined

^b^The total number of patients from each severity group does not equal 479 patients due to one patient for whom individual site was reported as “Not Done” at baseline in one of the bilateral sites, and therefore was not included in any of the severity groups

^c^*N1*, number of patients with non-missing data and baseline SPARCC > 0, if different from *N*

^d^*N1*, number of patients with non-missing data, if different from *N*

^e^*N1*, number of patients with non-missing data and baseline affected body surface area ≥ 3%, if different from *N*

BID, twice daily; BMI, body mass index; CPDAI, Composite Psoriatic Disease Activity in Psoriatic Arthritis; CRP, C-reactive protein; DAPSA, Disease Activity Index for Psoriatic Arthritis; DSS, Dactylitis Severity Score; FACIT-F, Functional Assessment of Chronic Illness Therapy-Fatigue; LEI, Leeds Enthesitis Index; *N*, total number of patients; *n*, number of patients applicable for each category; PASDAS, Psoriatic Arthritis Disease Activity Score; PsA, psoriatic arthritis; SD, standard deviation; SJC66, swollen joint count (out of 66 joints); SPARCC, Spondyloarthritis Research Consortium of Canada Enthesitis Index; TJC68, tender joint count (out of 68 joints); VAS, Visual Analog Scale

**Table 2:** Demographics and baseline disease characteristics of patients with SPARCC > 0 at baseline

	**Patients with SPARCC > 0 (*N* = 545)**					
	**Location**^**a**^			**Severity**^**b**^		
	**Upper sites only** ***N* = 64**	**Lower sites only** ***N* = 139**	**Upper and lower sites** ***N* = 342**	**1–2 sites** ***N* = 145**	** > 2– ≤ 5 sites** ***N* = 152**	** > 5 sites** ***N* = 242**
**Tofacitinib 5 mg BID**	***N* = 18**	***N* = 53**	***N* = 106**	***N* = 47**	***N* = 58**	***N* = 71**
**Tofacitinib 10 mg BID**	***N* = 23**	***N* = 45**	***N* = 123**	***N* = 45**	***N* = 48**	***N* = 95**
***Placebo***	***N* = *23***	***N* = *41***	***N* = *113***	***N* = *53***	***N* = *46***	***N* = *76***
Female, *n* (%)	7 (38.9)	17 (32.1)	64 (59.4)	19 (40.4)	24 (41.4)	43 (60.6)
	9 (39.1)	19 (42.2)	83 (67.5)	18 (40.0)	24 (50.0)	67 (70.5)
	*7 (30.4)*	*19 (46.3)*	*76 (67.3)*	*22 (41.5)*	*25 (54.3)*	*54 (71.1)*
Age, years, mean (SD)	53.3 (12.5)	43.9 (13.5)	50.8 (11.5)	45.4 (14.1)	49.1 (11.5)	51.3 (12.2)
	43.3 (14.3)	48.9 (13.0)	51.3 (10.5)	47.8 (12.9)	46.6 (13.2)	52.0 (10.1)
	*48.8 (12.7)*	*47.8 (13.8)*	*49.1 (12.7)*	*47.4 (12.8)*	*48.7 (14.7)*	*49.8 (11.9)*
Race, White, *n* (%)	17 (94.4)	51 (96.2)	100 (94.3)	46 (97.9)	54 (93.1)	67 (94.4)
	22 (95.7)	41 (91.1)	115 (93.5)	39 (86.7)	43 (89.6)	93 (97.9)
	*23 (100)*	*41 (100)*	*103 (91.2)*	*52 (98.1)*	*44 (95.7)*	*69 (90.8)*
BMI, kg/m^2^, mean (SD)	29.0 (7.2)	29.0 (5.9)	30.7 (6.6)	29.1 (7.1)	31.0 (7.0)	29.7 (5.5)
	27.7 (4.7)	28.9 (5.8)	30.8 (6.4)	28.3 (5.1)	29.3 (5.6)	31.0 (6.7)
	*29.4 (5.9)*	*29.8 (4.6)*	*29.4 (6.0)*	*29.0 (4.9)*	*29.3 (6.2)*	*30.1 (5.9)*
PsA duration, years, mean (SD)	9.2 (8.6)	6.9 (6.3)	8.6 (7.7)	7.8 (7.7)	8.0 (6.8)	8.6 (7.8)
	8.6 (8.5)	8.3 (7.6)	7.4 (6.2)	8.0 (8.3)	6.9 (6.4)	8.0 (6.3)
	*5.5 (4.6)*	*10.0 (9.2)*	*7.7 (6.2)*	*10.1 (8.8)*	*6.6 (4.6)*	*7.4 (6.3)*
CRP, mg/L, mean (SD)	17.6 (32.7)	19.7 (29.6)	10.4 (15.4)	16.8 (23.9)	15.3 (27.6)	11.0 (17.5)
	8.6 (8.4)	19.5 (32.1)	11.1 (20.7)	12.4 (18.0)	15.3 (27.5)	11.9 (23.5)
	*13.2 (33.6)*	*9.7 (10.2)*	*12.0 (20.8)*	*11.9 (23.5)*	*11.4 (20.8)*	*11.7 (19.8)*
LEI, mean (SD) [*N1*]^c^	1.5 (0.7) [12]	2.1 (1.1) [35]	3.2 (1.6) [101]	1.3 (0.5) [26]	2.2 (1.0) [50]	3.9 (1.4)
	1.7 (1.1) [15]	1.9 (1.1) [31]	3.8 (1.6) [113]	1.2 (0.4) [24]	2.5 (1.2) [40]	4.1 (1.5) [93]
	*1.6 (0.8) *[18]	*1.8 (0.9) *[24]	*3.2 (1.5) [107]*	*1.4 (0.6) [34]*	*2.2 (1.0) [42]*	*3.7 (1.4) [73]*
SPARCC, mean (SD)	2.3 (1.5)	3.0 (1.9)	7.2 (3.7)	1.6 (0.5)	4.0 (0.8)	9.1 (3.1)
	1.7 (1.2)	3.1 (2.4)	8.1 (3.7)	1.3 (0.5)	3.9 (0.7)	9.4 (3.2)
	*2.0 (1.2)*	*2.4 (1.4)*	*7.1 (3.4)*	*1.6 (0.5)*	*4.0 (0.8)*	*8.8 (2.8)*
Dactylitis presence, DSS > 0, *n* (%)	9 (50)	33 (62.3)	60 (56.6)	24 (51.1)	33 (56.9)	45 (63.4)
	12 (52.2)	30 (66.7)	65 (52.8)	25 (55.6)	27 (56.3)	53 (55.8)
	*10 (43.5)*	*23 (56.1)*	*61 (54.0)*	*25 (47.2)*	*24 (52.2)*	*44 (57.9)*
TJC68, mean (SD) [*N1*]^d^	13.7 (8.4) [6]	18.2 (9.4) [25]	25.2 (14.6) [50]	17.1 (10.3) [21]	20.8 (11.1) [31]	27.9 (16.0) [28]
	15.6 (6.9) [13]	18.3 (15.0) [19]	25.8 (13.2) [50]	15.1 (8.6) [26]	16.7 (12.6) [21]	31.8 (12.0) [33]
	*21.1 (15.7) *[13]	*15.0 (5.3) *[20]	*28.3 (15.9) [46]*	*15.3 (8.7) *[26]	*23.1 (13.5) *[16]	*31.0 (16.0) [35]*
SJC66, mean (SD) [*N1*]^d^	6.7 (3.6) [6]	12.4 (5.7) [25]	14.6 (11.3) [50]	11.6 (8.4) [21]	12.9 (7.4) [31]	15.4 (12.4) [28]
	10.5 (3.9) [13]	12.0 (8.6) [19]	13.2 (8.9) [50]	10.7 (6.2) [26]	9.4 (6.2) [21]	15.6 (9.8) [33]
	*12.8 (11.3) *[13]	*9.4 (5.6) *[20]	*13.7 (10.2) [46]*	*10.0 (6.9) *[26]	*13.6 (10.0) *[16]	*14.1 (10.9) [35]*
PASDAS, mean (SD) [*N1*]^d^	5.9 (1.4)	6.2 (1.2) [52]	6.4 (1.1) [102]	6.1 (1.3) [35]	6.3 (1.2) [45]	6.7 (1.0) [52]
	6.1 (0.9)	6.4 (1.0)	6.6 (1.1) [119]	6.1 (0.9) [35]	6.6 (1.1) [36]	6.9 (1.2) [65]
	*5.6 (1.2) *[22]	*6.0 (1.2) [40]*	*6.4 (1.1) [112]*	*5.9 (1.1) [39]*	*6.6 (1.0) [30]*	*6.8 (0.9) [56]*
DAPSA, mean (SD) [*N1*]^d^	44.5 (25.3)	42.2 (16.4)	52.4 (23.9) [105]	43.1 (22.6) [35]	45.2 (18.2) [45]	59.8 (25.8) [54]
	35.1 (12.9)	44.5 (23.9)	57.9 (25.8)	37.2 (14.7) [35]	46.5 (24.7) [36]	66.1 (27.1) [66]
	*38.4 (22.7)*	*36.0 (12.5)*	*51.7 (24.4)*	*37.1 (16.3) [39]*	*45.8 (20.0) [30]*	*60.2 (25.2) [56]*
CPDAI, mean (SD) [*N1*]^e^	8.9 (1.8) [13]	10.6 (2.3) [40]	11.3 (2.0) [62]	9.7 (2.2) [32]	10.4 (2.0) [28]	12.2 (1.9) [41]
	9.6 (2.5) [15]	11.0 (2.4) [30]	11.3 (2.3) [71]	9.6 (2.3) [17]	11.6 (1.9) [30]	12.1 (2.3) [45]
	*9.1 (2.3) *[16]	*10.5 (2.4) [33]*	*10.8 (2.5) [74]*	*10.1 (2.3) [31]*	*11.0 (2.4) *[26]	*11.9 (1.9) [39]*
FACIT-F total score, mean (SD) [*N1*]^d^	25.7 (14.0)	25.2 (11.1)	25.2 (11.1) [105]	26.2 (11.9)	25.8 (11.6)	24.1 (10.9) [70]
	31.2 (10.0)	25.6 (9.3)	25.0 (10.4)	29.8 (10.3)	24.9 (9.4)	24.4 (10.2)
	*31.7 (11.5)*	*25.0 (9.7)*	*27.3 (10.4)*	*28.3 (10.5)*	*29.0 (11.5)*	*25.8 (9.8)*
Arthritis pain (VAS), mean (SD) [*N1*]^d^	62.8 (24.0)	59.0 (22.2)	56.0 (22.6) [105]	60.3 (25.3)	54.3 (22.5)	58.4 (20.9) [70]
	54.5 (23.3)	58.6 (22.8)	60.4 (20.7)	57.6 (21.6)	56.8 (22.8)	61.1 (21.0)
	*44.2 (21.2)*	*55.2 (23.7)*	*57.3 (24.3)*	*48.7 (23.0)*	*58.1 (25.6)*	*58.3 (22.6)*

^a^Each site was assessed bilaterally, and results were combined

^b^The total number of patients from each severity group does not equal 545 patients due to six patients for whom individual site was reported as “Not Done” at baseline in one of the bilateral sites, and therefore were not included in any of the severity groups

^c^*N1*, number of patients with non-missing data and baseline LEI > 0, if different from *N*

^d^*N1*, number of patients with non-missing data, if different from *N*

^e^*N1*, number of patients with non-missing data and baseline affected body surface area ≥ 3%, if different from *N*

BID, twice daily; BMI, body mass index; CPDAI Composite Psoriatic Disease Activity in Psoriatic Arthritis; CRP, C-reactive protein; DAPSA, Disease Activity Index for Psoriatic Arthritis; DSS, Dactylitis Severity Score; FACIT-F, Functional Assessment of Chronic Illness Therapy-Fatigue; LEI, Leeds Enthesitis Index; *N*, total number of patients; *n*, number of patients applicable for each category; PASDAS, Psoriatic Arthritis Disease Activity Score; PsA, psoriatic arthritis; SD, standard deviation; SJC66, swollen joint count (out of 66 joints); SPARCC, Spondyloarthritis Research Consortium of Canada Enthesitis Index; TJC68, tender joint count (out of 68 joints); VAS, Visual Analog Scale

### Improvements in patients with enthesitis at baseline

**Fig. 2:**
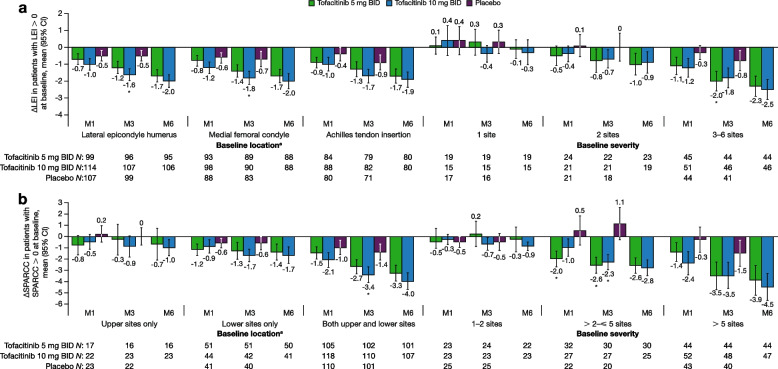
Change from baseline in LEI/SPARCC (patients with LEI > 0/SPARCC > 0 at baseline). *Indicates a comparison where the 95% CI for tofacitinib does not overlap with the 95% CI for placebo. ^a^Each site was assessed bilaterally, and results were combined. Δ, change from baseline; BID, twice daily; CI, confidence interval; LEI, Leeds Enthesitis Index; M, month; *N*, total number of patients with LEI > 0 or SPARCC > 0 in that particular location or number of affected sites at baseline; SPARCC, Spondyloarthritis Research Consortium of Canada Enthesitis Index

Compared with placebo, improvements from baseline in LEI scores were greater with tofacitinib 10 mg BID at month 3 in patients with enthesitis at the lateral epicondyle humerus or medial femoral condyle, and greater with tofacitinib 5 mg BID in patients with 3–6 affected LEI sites at baseline (Fig. 2a). Tofacitinib treatment was associated with greater improvements in SPARCC scores vs placebo in patients with enthesitis in both upper and lower sites at baseline (10 mg BID dose at month 3) and in patients with > 2– ≤ 5 affected SPARCC sites at baseline (5 mg BID dose at month 1 and both dose groups at month 3; Fig. 2b). For both enthesitis indices, improvements with tofacitinib were maintained and continued through month 6, regardless of baseline enthesitis location or severity, except in patients with 1 affected LEI site or 1–2 affected SPARCC sites at baseline (Fig. 2).

#### Trajectory of enthesitis over time in patients with enthesitis at baseline

**Fig. 3:**
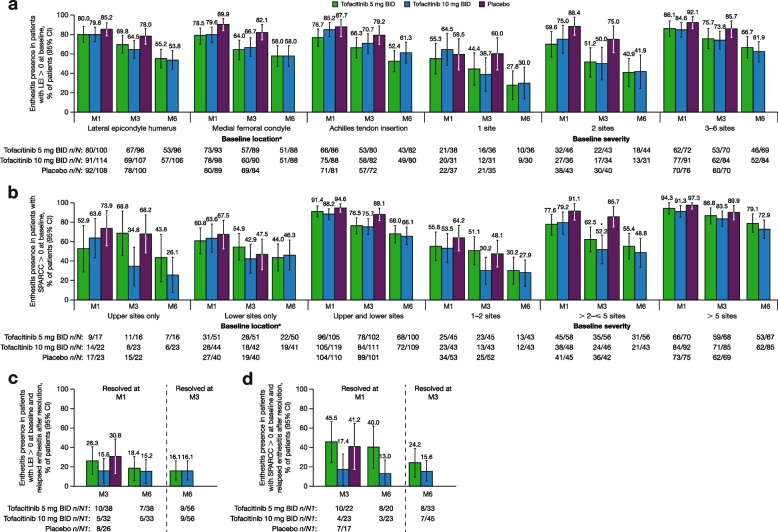
Enthesitis presences/relapse of resolved enthesitis (patients with LEI > 0/SPARCC > 0 at baseline). *Indicates a comparison where the 95% CI for tofacitinib does not overlap with the 95% CI for placebo. ^a^Each site was assessed bilaterally, and results were combined. BID, twice daily; CI, confidence interval; LEI, Leeds Enthesitis Index; M, month; *N*, total number of patients with LEI > 0 or SPARCC > 0 in that particular location or number of affected sites at baseline; *N1*, total number of patients with LEI > 0 or SPARCC > 0 at baseline and with LEI = 0 or SPARCC = 0 at months 1 or 3; *n*, number of affected patients; SPARCC, Spondyloarthritis Research Consortium of Canada Enthesitis Index

Regardless of baseline location or severity, the proportion of patients with enthesitis (LEI > 0 or SPARCC > 0) generally decreased over time with tofacitinib treatment (Fig. 3a, b). At month 3, the proportion of patients with > 2– ≤ 5 affected SPARCC sites at baseline who had enthesitis was lower with tofacitinib 10 mg BID vs placebo (Fig. 3b).

At month 6, the proportions of tofacitinib-treated patients with enthesitis assessed by LEI were reduced by 40–50% across baseline locations (Fig. 3a). Similarly, at month 6, the proportions of tofacitinib-treated patients with enthesitis assessed by SPARCC were reduced by over 50% in patients with enthesitis in upper or lower sites only at baseline. Approximately two-thirds of patients with enthesitis in both upper and lower sites at baseline still had enthesitis after 6 months (Fig. 3b). Whether assessed by LEI or SPARCC, enthesitis presence at month 6 was more prominent in patients with greater vs lower severity at baseline (Fig. 3a, b).

For patients with LEI > 0 at baseline whose enthesitis had resolved (LEI = 0) at month 1, 26.3%, 15.6%, and 30.8% of patients treated with tofacitinib 5 mg BID, tofacitinib 10 mg BID, and placebo, respectively, had relapsed at month 3 (Fig. 3c). At month 6, relapsed enthesitis was observed in 18.4% and 15.2% of patients treated with tofacitinib 5 mg or 10 mg BID, respectively, who had resolved enthesitis at month 1. In patients who had resolved enthesitis at month 3, 16.1% of patients in each tofacitinib group had relapsed at month 6 (Fig. 3c).

At month 3, for patients with SPARCC > 0 at baseline who had resolved enthesitis (SPARCC = 0) at month 1, 45.5%, 17.4%, and 41.2% of patients treated with tofacitinib 5 mg BID, tofacitinib 10 mg BID, and placebo, respectively, had relapsed (Fig. 3d). At month 6, relapsed enthesitis was observed in 40.0% and 13.0% of patients treated with tofacitinib 5 and 10 mg BID, respectively, who had resolved enthesitis at month 1. Among patients who had resolved enthesitis at month 3, 24.2% and 15.6% of patients relapsed at month 6 (Fig. 3d).

### De novo enthesitis in patients without enthesitis at baseline

**Fig. 4:**
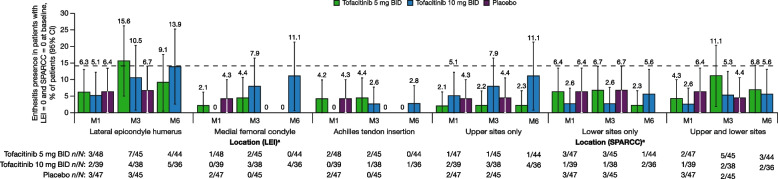
De novo enthesitis development (patients with LEI = 0 and SPARCC = 0 at baseline). The dashed line indicates < 14% of patients without enthesitis (LEI = 0 and SPARCC = 0) at baseline with enthesitis at month 6. ^a^Each site was assessed bilaterally, and results were combined. BID, twice daily; CI, confidence interval; LEI, Leeds Enthesitis Index; M, month; *N*, total number of patients with LEI = 0 and SPARCC = 0 at baseline; *n*, number of affected patients; SPARCC, Spondyloarthritis Research Consortium of Canada Enthesitis Index

For patients without enthesitis (LEI = 0 and SPARCC = 0) at baseline, ≤ 6.4% and ≤ 15.6% of patients receiving tofacitinib or placebo developed enthesitis across locations (LEI and SPARCC) at months 1 and 3, respectively (Fig. 4). At month 6, among the 44 patients without enthesitis at baseline in the tofacitinib 5 mg BID group, 4 (9.1%) patients had developed enthesitis at the lateral epicondyle humerus as assessed by LEI; 1 (2.3%), 1 (2.3%), and 3 (6.8%) patients had developed enthesitis at the upper sites only, lower sites only, and both upper and lower sites, respectively, as assessed by SPARCC (Fig. 4). At month 6, among the 36 patients without enthesitis at baseline in the tofacitinib 10 mg BID group, 5 (13.9%), 4 (11.1%), and 1 (2.8%) patients had developed enthesitis at the lateral epicondyle humerus, medial femoral condyle, and Achilles tendon insertion, respectively, as assessed by LEI; 4 (11.1%), 2 (5.6%), and 2 (5.6%) patients had developed enthesitis at the upper sites only, lower sites only, and both upper and lower sites, respectively, as assessed by SPARCC (Fig. 4).

At months 1 and 3, the proportions of patients without enthesitis at baseline who developed de novo enthesitis were similar with tofacitinib and placebo, regardless of enthesitis location (LEI and SPARCC; Fig. 4). Across locations assessed by LEI, development of enthesitis at the lateral epicondyle humerus was observed at all time points across treatments (Fig. 4).

### Disease activity in patients with or without enthesitis at baseline

Overall, regardless of baseline enthesitis location or severity, the proportion of tofacitinib-treated patients with enthesitis (LEI > 0 or SPARCC > 0) at baseline who achieved LDA or remission increased over time, though there were some fluctuations from months 1 to 6 (Supplementary Figs. 1, 2 [see Additional file 1]).

Among patients with LEI > 0 at baseline, at month 3, MDA (≥ 5/7 criteria) rates were greater with tofacitinib vs placebo in patients with enthesitis at the lateral epicondyle humerus (5 mg BID only) and medial femoral condyle at baseline (both 5 and 10 mg BID; Supplementary Fig. 1a [see Additional file 1]). In patients with enthesitis at the medial femoral condyle at baseline, at month 1, PASDAS LDA (> 1.9– < 3.2) rates were greater with both tofacitinib doses vs placebo; at month 3, greater rates were observed with tofacitinib 10 mg BID in patients with enthesitis at the lateral epicondyle humerus and medial femoral condyle at baseline (Supplementary Fig. 1b [see Additional file 1]).

Among patients with SPARCC > 0 at baseline, at month 1, the proportion of patients who achieved MDA was greater with tofacitinib 10 mg BID vs placebo in patients with enthesitis at the lower sites only at baseline; at month 3, a greater proportion of patients with enthesitis at the upper and lower sites at baseline achieved MDA with tofacitinib 5 mg BID vs placebo (Supplementary Fig. 2a [see Additional file 1]). PASDAS/DAPSA LDA (> 1.9– < 3.2/ > 4– ≤ 14, respectively) rates at month 3 were greater in patients with enthesitis in upper sites only with tofacitinib 10 mg BID vs placebo (Supplementary Fig. 2b, c [see Additional file 1]). A greater PASDAS LDA rate at month 3 was also observed in patients with enthesitis in upper and lower sites at baseline with tofacitinib 10 mg BID vs placebo (Supplementary Fig. 2b [see Additional file 1]).

Across baseline enthesitis severity (LEI or SPARCC), some differences in MDA (≥ 5/7 criteria) or PASDAS/DAPSA LDA (> 1.9– < 3.2/ > 4– ≤ 14, respectively) rates between patients treated with tofacitinib vs placebo were observed at months 1 and 3 (Supplementary Fig. 1a–c, 2a–c [see Additional file 1]).

Through month 3, remission rates were similar in patients with enthesitis at baseline treated with tofacitinib and placebo, except CPDAI remission (≤ 2) rates were higher at month 3 in patients with 2 affected LEI sites or ≥ 2– > 5 affected SPARCC sites at baseline who received tofacitinib 10 mg BID (Supplementary Figs. 1e–h, 2e–h [see Additional file 1]).

The proportions of patients without enthesitis (LEI = 0 and SPARCC = 0) at baseline reporting LDA or remission were similar across treatments (Supplementary Fig. 3a–h [see Additional file 1]); however, at month 3, PASDAS LDA and near remission (> 1.9– < 3.2 and ≤ 1.9, respectively) rates were higher with tofacitinib 10 mg BID and tofacitinib 5 mg BID, respectively, vs placebo (Supplementary Fig. 3b, f [see Additional file 1]).

### Patient-reported outcomes in patients with or without enthesitis at baseline

Mean FACIT-F total scores for patients with enthesitis (LEI > 0 or SPARCC > 0) at baseline receiving tofacitinib and placebo are shown in Supplementary Fig. 4 (see Additional file 1).

At months 1 and 3, among patients with LEI > 0 at baseline, improvements in mean arthritis pain (VAS) scores were greater with tofacitinib 10 mg BID vs placebo across locations, except at month 1 in patients with enthesitis at the Achilles tendon insertion (Supplementary Fig. 4c [see Additional file 1]). Across enthesitis severity at baseline, greater improvements in mean arthritis pain (VAS) scores were observed with tofacitinib 10 mg BID vs placebo in patients with 2 affected sites (months 1 and 3) and 3–6 sites (month 1) at baseline (Supplementary Fig. 4c [see Additional file 1]). Improvements in mean arthritis pain (VAS) scores were greater with tofacitinib 5 mg BID vs placebo at months 1 and 3 in patients with enthesitis at the medial femoral condyle at baseline and at month 3 in patients with 3–6 affected sites at baseline (Supplementary Fig. 4c [see Additional file 1]). Among patients with SPARCC > 0 at baseline, at months 1 and 3, both tofacitinib doses were associated with greater improvements in mean arthritis pain (VAS) scores in those with enthesitis in both upper and lower sites at baseline; greater improvements with tofacitinib 10 mg BID vs placebo were also observed at month 3 in patients with enthesitis at the lower sites only at baseline (Supplementary Fig. 4d [see Additional file 1]). Patients with > 5 affected SPARCC sites at baseline had greater improvements in mean arthritis pain (VAS) scores with tofacitinib vs placebo at month 1 (both doses) and 3 (5 mg BID dose only); significant differences were also observed with tofacitinib 10 mg BID at months 1 and 3 in patients with 1–2 affected sites at baseline and with tofacitinib 5 mg BID at month 3 in patients with > 2– ≤ 5 affected sites at baseline (Supplementary Fig. 4d [see Additional file 1]). Overall, improvements were maintained through month 6.

In patients without enthesitis (LEI = 0 and SPARCC = 0) at baseline, mean FACIT-F total and arthritis pain (VAS) scores through month 3 were similar across treatments (Supplementary Fig. 4e, f [see Additional file 1]).

PRO scores improved over time with tofacitinib treatment and were maintained to month 6 in patients with enthesitis (LEI > 0 or SPARCC > 0), regardless of baseline location and severity, and were maintained in patients without enthesitis (LEI = 0 and SPARCC = 0) at baseline (Supplementary Fig. 4a–f [see Additional file 1]).

Multivariable linear regression analyses demonstrated that baseline enthesitis severity assessed by SPARCC was significantly associated with change from baseline in FACIT-F total score at months 3 (*p* = 0.0004) and 6 (*p* = 0.0010) in the pooled tofacitinib group. The estimates (95% CI) for the specific comparison of > 5 vs 0 affected sites were -3.3 (-5.7, -0.9; *p* = 0.0064) and -3.7 (-6.2, -1.1; *p* = 0.0050) at months 3 and 6, respectively.

When each tofacitinib dose was assessed individually, baseline enthesitis severity assessed by SPARCC was significantly associated with change from baseline in FACIT-F total score with tofacitinib 10 mg BID at month 3 (*p* = 0.0042) and with tofacitinib 5 mg BID at month 6 (*p* = 0.0103).

Baseline enthesitis location and severity assessed by LEI and baseline enthesitis location assessed by SPARCC were not significantly associated with changes from baseline in FACIT-F total score at months 3 and 6, irrespective of tofacitinib dose.

Similarly, baseline enthesitis location and severity assessed by LEI and baseline enthesitis location assessed by SPARCC were not associated with changes from baseline in arthritis pain score in the pooled tofacitinib dose group. However, baseline enthesitis severity assessed by SPARCC was significantly associated with change from baseline in arthritis pain score at month 3 (*p* < 0.0001) and 6 (*p* = 0.0001) in the pooled tofacitinib group. The estimates (95% CI) for the specific comparison of > 5 vs 0 affected sites were 12.3 (6.8, 17.8; *p* < 0.0001) and 11.0 (4.6, 17.3; *p* = 0.0007) at months 3 and 6, respectively.

When each tofacitinib dose was assessed individually, enthesitis presence, assessed by LEI, at the lateral epicondyle humerus and Achilles tendon insertion in patients treated with tofacitinib 5 mg BID was significantly associated with change from baseline in arthritis pain score at month 3 (7.8 [1.2, 14.4], *p* = 0.0205 and 9.6 [2.8, 16.5], *p* = 0.0063, respectively). Baseline enthesitis severity assessed by SPARCC was significantly associated with change from baseline in arthritis pain score in patients treated with tofacitinib 10 mg BID at month 3 (*p* = 0.0045) and with tofacitinib 5 and 10 mg BID at month 6 (*p* = 0.0120 and *p* = 0.0065, respectively).

There were no significant associations between baseline enthesitis location assessed by SPARCC and change from baseline in arthritis pain score when each tofacitinib dose was assessed individually.

## Discussion

In phase 3 studies of patients with PsA (OPAL Broaden and OPAL Beyond), greater improvements in enthesitis and more frequent enthesitis resolution were reported at month 3 with tofacitinib vs placebo [9, 10]. This post hoc analysis explored the effects of tofacitinib on enthesitis in patients with enthesitis at baseline, by location or severity, and compared outcomes among patients with vs without enthesitis at baseline.

At month 3, improvements in LEI were greater with tofacitinib 10 mg BID vs placebo in patients with enthesitis at the lateral epicondyle humerus or medial femoral condyle at baseline (10 mg BID) and in patients with 3–6 affected sites (5 mg BID); while greater improvements in SPARCC were observed with tofacitinib vs placebo in patients with enthesitis in both upper and lower sites at baseline (10 mg BID at month 3) and in patients with > 2– ≤ 5 affected SPARCC sites at baseline (5 mg BID at month 1 and both doses at month 3). Improvements from baseline in enthesitis in tofacitinib-treated patients were observed as early as month 1 and maintained to month 6; greatest improvements were seen in patients with greatest severity at baseline. Regardless of baseline enthesitis location or severity, enthesitis presence generally decreased over time with tofacitinib treatment. At month 6, relapse of previously resolved enthesitis was observed in ≤ 40% of tofacitinib-treated patients with enthesitis at baseline. Disease activity remission rates and fatigue scores were generally similar across treatments. Tofacitinib treatment was associated with greater LDA rates or improvements from baseline in pain scores compared with placebo, though fluctuations were observed over time.

Through month 6, presence of enthesitis, as assessed by LEI, was similar across baseline enthesitis location, indicating that no site was at a greater risk for enthesitis. Enthesitis by SPARCC was more common in patients with enthesitis in both upper and lower sites at baseline. These differences in treatment response are aligned with results from a previous analysis of the US CorEvitas (formerly Corrona) PsA/Spondylarthritis (SpA) Registry, where impact of enthesitis, assessed by SPARCC, on disease activity was highest in patients with enthesitis at both upper and lower sites, followed by lower sites only and upper sites only [16]. In our study, longer-term enthesitis was less frequent in patients with lower vs greater severity at baseline.

Multivariable linear regression analyses demonstrated that enthesitis at the lateral epicondyle humerus and Achilles tendon insertion, as assessed by LEI, was significantly associated with change from baseline in arthritis pain scores at month 3 following treatment with tofacitinib 5 mg BID, suggesting that these sites may have a greater role on disease activity. When assessed by SPARCC, baseline enthesitis severity (specifically, > 5 vs 0 affected sites comparison) was significantly associated with change from baseline in FACIT-F total and arthritis pain scores in the pooled (months 3 and 6) and individual tofacitinib groups (tofacitinib 5 mg BID: month 6 for FACIT-F total and arthritis pain scores; tofacitinib 10 mg BID: month 3 for FACIT-F total scores, months 3 and 6 for arthritis pain scores); however, no significant association between baseline enthesitis location and change from baseline in FACIT-F total and arthritis pain scores was identified in the pooled or individual tofacitinib groups. This finding suggests that enthesitis severity, and not location, may have a greater impact on treatment response for patients; however, in the US CorEvitas PsA/SpA Registry analysis, enthesitis presence in lower or both upper and lower sites was significantly associated with greater pain and fatigue score [16].

In this analysis, development of de novo enthesitis was low and similar for tofacitinib and placebo. Interestingly, when assessed by LEI, emerging enthesitis at the lateral epicondyle humerus was observed at all time points across treatments. It should be noted that enthesopathy at the lateral epicondyle humerus is not infrequent in the general population [17, 18], and tenderness at this site may not reflect new onset inflammatory enthesitis related to PsA. As such, the lateral epicondyle humerus may be less reliable in assessing treatment response although being commonly involved in PsA and tenderness at this site only partially links to sonographic enthesitis [19].

The effects of other PsA treatments on individual enthesitis sites or differing enthesitis severities have been reported previously. Efficacy of ixekizumab on enthesitis resolution rates was generally consistent across locations when assessed by LEI [20]. Differences in treatment response based on enthesitis location with adalimumab vs placebo were reported in a post hoc analysis of a phase 3 study that included 165 patients with peripheral SpA; at the Achilles tendon and lateral epicondyle humerus, the number of enthesitis sites was reduced by over 50%, and significantly greater resolution rates and less development of de novo enthesitis were observed in patients treated with adalimumab vs placebo [21]. At week 24, greatest improvement in LEI with guselkumab vs placebo was observed in patients with severe enthesitis at baseline; however, by week 52, resolution was more common in patients with mild or moderate enthesitis at baseline [22]. A pooled analysis of two phase 3 studies in patients with PsA treated with secukinumab demonstrated that enthesitis resolution at week 104 was less prominent in patients with more vs less severe enthesitis at baseline [23].

Limitations of this study include the post hoc nature of the analysis, low patient numbers in several enthesitis location or severity subgroups, and lack of adjustment for multiple comparisons. Additionally, comparisons with placebo were restricted to 3 months, and the effects of tofacitinib on enthesitis were only assessed up to month 6, consistent with the original study designs. The clinical enthesitis assessments used also have known limitations. While LEI was designed specifically to assess enthesitis in patients with PsA [12], and SPARCC has been used widely in patients with SpA [24], to our knowledge, intra-observer reliability for LEI and SPARCC has not been studied, and strong inter-observer reliability was demonstrated only in patients with PsA with axial involvement [25]. Furthermore, clinical enthesitis assessment at peripheral sites by physical examination may not accurately assess true inflammation in the entheses, compared with advanced imaging techniques such as ultrasound or magnetic resonance imaging [26, 27]. Central pain sensitization, manifested as widespread allodynia, may be associated with chronic inflammatory musculoskeletal disorders; therefore, tenderness may be elicited on physical examination, even when no inflammation is present and vice versa [28]. When inflammation is abrogated, regardless of its mechanism, allodynia may diminish [26] and subsequently reflect as an improvement in the clinical “enthesitis” score, despite any actual improvement in inflammation at the entheses [29]. This effect may be more prominent in enthesitis sites near the fibromyalgia points, including the elbows and knees.

## Conclusion

This post hoc analysis demonstrated greater improvements from baseline in enthesitis and disease activity measures in patients with PsA receiving tofacitinib vs placebo across several enthesitis locations and with varying enthesitis severity at baseline. An association between enthesitis at the lateral epicondyle humerus and Achilles tendon insertion and improvement in pain, and an association between enthesitis severity (assessed by SPARCC) and improvements in fatigue and pain were identified. These findings support tofacitinib as a treatment option for patients with PsA with enthesitis and affirm the importance of addressing enthesitis in the PsA treatment paradigm.

### Supplementary Information

**Additional file 1:** **Supplementary Table 1.** Components of the disease activity measures used. **Supplementary Table 2.** Demographics and baseline disease characteristics of patients with LEI = 0 and SPARCC = 0 at baseline. **Supplementary Fig. 1.** LDA and remission rates, based on MDA or VLDA, PASDAS, DAPSA, and CPDAI criteria (patients with LEI > 0 at baseline). **Supplementary Fig. 2.** LDA and remission rates, based on MDA or VLDA, PASDAS, DAPSA, and CPDAI criteria (patients with SPARCC > 0 at baseline). **Supplementary Fig. 3.** LDA and remission rates, based on MDA or VLDA, PASDAS, DAPSA, and CPDAI criteria (patients with LEI = 0 and SPARCC = 0 at baseline). **Supplementary Fig. 4.** FACIT-F total and arthritis pain (VAS) scores (patients with LEI > 0/SPARCC > 0/LEI = 0 and SPARCC = 0 at baseline).

## Data Availability

Upon request, and subject to review, Pfizer will provide the data that support the findings of this study. Subject to certain criteria, conditions and exceptions, Pfizer may also provide access to the related individual de-identified participant data. See https://www.pfizer.com/science/clinical-trials/trial-data-and-results for more information.

## Abbreviations

BID: Twice daily
BMI: Body mass index
CI: Confidence interval
CPDAI: Composite Psoriatic Disease Activity in Psoriatic Arthritis
CRP: C-reactive protein
csDMARD: Conventional synthetic disease-modifying antirheumatic drug
DAPSA: Disease Activity Index for Psoriatic Arthritis
DSS: Dactylitis Severity Score
FACIT-F: Functional Assessment of Chronic Illness Therapy-Fatigue
LDA: Low disease activity
LEI: Leeds Enthesitis Index
MDA: Minimal disease activity
PASDAS: Psoriatic Arthritis Disease Activity Score
PROs: Patient-reported outcomes
PsA: Psoriatic arthritis
SD: Standard deviation
SJC: Swollen joint count
SPARCC: Spondyloarthritis Research Consortium of Canada Enthesitis Index
TJC: Tender joint count
TNFi: Tumor necrosis factor inhibitor
VAS: Visual Analog Scale
