# Supplementary material for: Efficacy of tofacitinib on enthesitis in patients with active psoriatic arthritis: analysis of pooled data from two phase 3 studies
Source: Arthritis Res Ther. 2023 Aug 22;25:153. doi: 10.1186/s13075-023-03108-5 (PMC10464128; doi:10.1186/s13075-023-03108-5)
Supplement: Supplementary file 1 — Additional file 1: Supplementary Table 1. Components of the disease activity measures used. Supplementary Table 2. Demographics and baseline disease characteristics of patients with LEI = 0 and SPARCC = 0 at baseline. Supplementary Fig. 1. LDA and remission rates, based on MDA or VLDA, PASDAS, DAPSA, and CPDAI criteria (patients with LEI > 0 at baseline). Supplementary Fig. 2. LDA and remission rates, based on MDA or VLDA, PASDAS, DAPSA, and CPDAI criteria (patients with SPARCC > 0 at baseline). Supplementary Fig. 3. LDA and remission rates, based on MDA or VLDA, PASDAS, DAPSA, and CPDAI criteria (patients with LEI = 0 and SPARCC = 0 at baseline). Supplementary Fig. 4. FACIT-F total and arthritis pain (VAS) scores (patients with LEI > 0/SPARCC > 0/LEI = 0 and SPARCC = 0 at baseline). [file 13075_2023_3108_MOESM1_ESM.pdf]

Additional file 1

Supplementary Table 1. Components of the disease activity measures used

Disease activity measures	Components	Computation	Cutoff points	
			LDA	Remission
MDA [1]	TJC ≤ 1	NA	≥ 5/7[a]	NA
VLDA [2]	SJC ≤ 1<br>PASI ≤ 1 or an affected BSA ≤ 3%<br>Patient arthritis pain (VAS) ≤ 15 mm<br>Patient global assessment of arthritis (VAS) ≤ 20 mm<br>HAQ-DI ≤ 0.5<br>Tender entheseal points (using LEI) ≤ 1		NA	7/7
PASDAS (range 0–10) [3]	TJC68<br>SJC66<br>Patient's global assessment (VAS)<br>Physician's global assessment (VAS)	PASDAS = {(0.18 × √physician's global assessment) + (0.159 × √patient's global assessment) – (0.253 × √SF-36 PCS) + [0.101 × ln(SJC66 + 1)] + [0.048 × ln(TJC68 + 1)] + [0.23 × ln(LEI score count + 1)] +	> 1.9–< 3.2 [4]	≤ 1.9[b] [2]

	LEI score<br>Tender dactylitis digit score<br>SF-36 PCS<br>CRP (mg/L)	[0.377 ln(tender dactylitis count + 1)] + [0.102 × ln(CRP + 1)] + 2} × 1.5		
DAPSA [5]	TJC68<br>SJC66<br>Patient's global assessment of arthritis (VAS)<br>Patient's pain assessment (VAS)<br>CRP (mg/dL)	DAPSA = TJC68 + SJC66 + CRP + patient's pain assessment + patient's global assessment of arthritis	> 4–≤ 14 [6]	≤ 4
CPDAI (range 0–15) [7]	TJC68<br>SJC66<br>HAQ<br>PASI<br>DLQI<br>LEI score	Disease activity under the following domains were graded as none (0), mild (1), moderate (2), or severe (3):<br>Peripheral arthritis<br>1. None: no involvement	> 2–≤ 4 [8]	≤ 2 [2]

Dactylitis digit score<br>BASDAI<br>ASQoL	2. Mild: TJC/SJC ≤ 4; normal function (HAQ < 0.5)<br>3. Moderate: TJC/SJC ≤ 4 but function impaired or TJC/SJC > 4 and normal function<br>4. Severe: TJC/SJC > 4 and function impaired<br>Skin disease<br>1. None: no involvement<br>2. Mild: PASI ≤ 10 and DLQI ≤ 10<br>3. Moderate: PASI ≤ 10 but DLQI > 10 or PASI > 10 and DLQI ≤ 10<br>4. Severe: PASI > 10 and DLQI > 10<br>Enthesitis<br>1. None: no involvement<br>2. Mild: LEI ≤ 3 sites; normal function (HAQ < 0.5)

3. Moderate: LEI ≤ 3 sites but function impaired or LEI > 3 and normal function
4. Severe: LEI > 3 and function impaired

Dactylitis

1. None: no involvement
2. Mild: ≤ 3 digits; normal function (HAQ < 0.5)
3. Moderate: ≤ 3 digits but function impaired or > 3 digits and normal function
4. Severe: > 3 digits and function impaired

Spinal disease

1. None: no involvement
2. Mild: BASDAI ≤ 4; normal function (ASQoL ≤ 6)

	3. Moderate: BASDAI ≤ 4 but function impaired or BASDAI > 4 and normal function<br>4. Severe: BASDAI > 4 and function impaired

[a]Included patients who fulfilled 6/7 and 7/7 MDA criteria

[b]PASDAS near remission

ASQoL, Ankylosing Spondylitis Quality of Life; BASDAI, Bath Ankylosing Spondylitis Disease Activity Index; BSA, body surface area; CPDAI, Composite Psoriatic Disease Activity Index; CRP, C-reactive protein; DAPSA, Disease Activity Index for Psoriatic Arthritis; DLQI, Dermatology Life Quality Index; HAQ-DI, Health Assessment Questionnaire-Disability Index; LEI, Leeds Enthesitis Index; LDA, low disease activity; MDA, minimal disease activity; NA, not available; PASDAS, Psoriatic Arthritis Disease Activity Score; PASI, Psoriasis Area and Severity Index; SF-36 PCS, Short Form-36 Health Survey physical component summary; SJC66, swollen joint count (out of 66 joints); TJC, tender joint count (out of 68 joints); VAS, Visual Analog Scale; VLDA, very low disease activity

Supplementary Table 2. Demographics and baseline disease characteristics of patients with LEI = 0 and SPARCC = 0 at baseline

	**Patients with LEI = 0 and SPARCC = 0 (*N* =136)**
Tofacitinib 5 mg BID	*N* = 49
Tofacitinib 10 mg BID	*N* = 39
Placebo	*N = 48*
Female, *n* (%)	26 (53.1)
	22 (56.4)
	27 (56.3)
Age, years, mean (SD)	50.8 (12.2)
	47.2 (10.8)
	46.9 (10.2)
Race, White, *n* (%)	46 (93.9)
	38 (97.4)
	45 (93.8)
BMI, kg/m^2, mean (SD)	29.4 (6.0)
	31.3 (6.6)
	27.6 (5.3)
PsA duration, mean (SD)	10.0 (9.1)
	6.1 (5.3)
	7.6 (8.4)
CRP, mg/L, mean (SD)	7.1 (9.9)
	7.6 (9.7)
	9.8 (18.5)

Dactylitis presence, DSS > 0, *n* (%)	18 (36.7)<br>16 (41.0)<br>*20 (41.7)*
TJC68, mean (SD) [*N1*][a]	13.4 (6.6) [17]<br>12.0 (5.4) [20]<br>*11.2 (6.4) [25]*
SJC66, mean (SD) [*N1*][a]	7.8 (3.0) [17]<br>8.1 (3.2) [20]<br>*8.6 (4.9) [25]*
PASDAS, mean (SD) [*N1*][a]	5.3 (1.0) [46]<br>5.1 (1.0) [38]<br>*5.2 (1.1) [48]*
DAPSA, mean (SD)	33.2 (15.4)<br>30.2 (11.5)<br>*29.2 (11.5)*
CPDAI, mean (SD) [*N1*][b]	7.6 (1.8) [36]<br>8.0 (2.1) [27]<br>*7.3 (2.1) [37]*
FACIT-F total score, mean (SD)	30.6 (10.5)<br>31.9 (10.0)<br>*30.7 (10.0)*
Arthritis pain (VAS), mean (SD)	49.3 (27.1)<br>45.9 (20.7)<br>*51.0 (27.2)*

[a]*N1* = number of patients with non-missing data, if different from *N*

[b]*N1* = number of patients with non-missing data and baseline affected BSA ≥3%, if different from *N*

BID, twice daily; BMI, body mass index; BSA, body surface area; CPDAI, Composite Psoriatic Disease Activity in Psoriatic Arthritis; CRP, C-reactive protein; DAPSA, Disease Activity Index for Psoriatic Arthritis; DSS, Dactylitis Severity Score; FACIT-F, Functional Assessment of Chronic Illness Therapy-Fatigue; LEI, Leeds Enthesitis Index; *N*, total number of patients; *n*, number of patients applicable for each category; PASDAS, Psoriatic Arthritis Disease Activity Score; PsA, psoriatic arthritis; SD, standard deviation; SJC66, swollen joint count (out of 66 joints); SPARCC, Spondyloarthritis Research Consortium of Canada Enthesitis Index; TJC68, tender joint count (out of 68 joints); VAS, Visual Analog Scale

Supplementary Fig. 1 LDA and remission rates, based on MDA or VLDA, PASDAS[a], DAPSA, and CPDAI[b] criteria (patients with LEI > 0 at baseline)

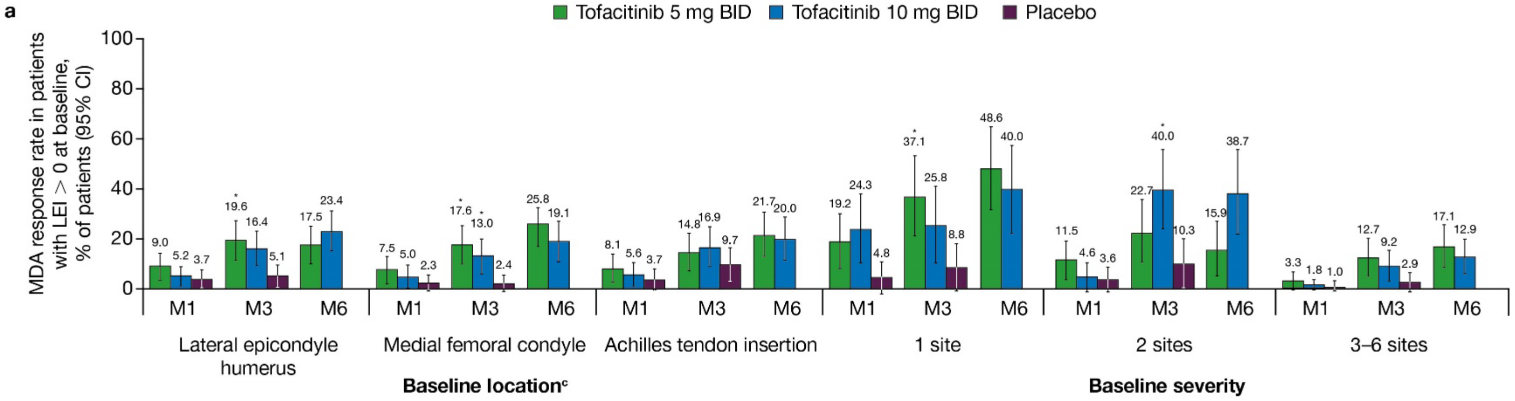

	M1	M3	M6	M1	M3	M6	M1	M3	M6
Tofacitinib 5 mg BID *n/N*:	9/100	19/97	17/97	7/93	16/91	23/89	7/86	12/81	18/83
Tofacitinib 10 mg BID *n/N*:	6/116	18/110	25/107	5/100	12/92	17/89	5/90	14/83	16/80
Placebo *n/N*:	4/108	5/98		2/89	2/84		3/81	7/72	

	M1	M3	M6	M1	M3	M6	M1	M3	M6
Tofacitinib 5 mg BID *n/N*:	9/47	13/35	17/35	7/61	10/44	7/44	3/91	9/71	12/70
Tofacitinib 10 mg BID *n/N*:	9/37	8/31	12/30	2/44	14/35	12/31	2/113	8/87	11/85
Placebo *n/N*:	2/42	3/34		2/56	4/39		1/96	2/70	

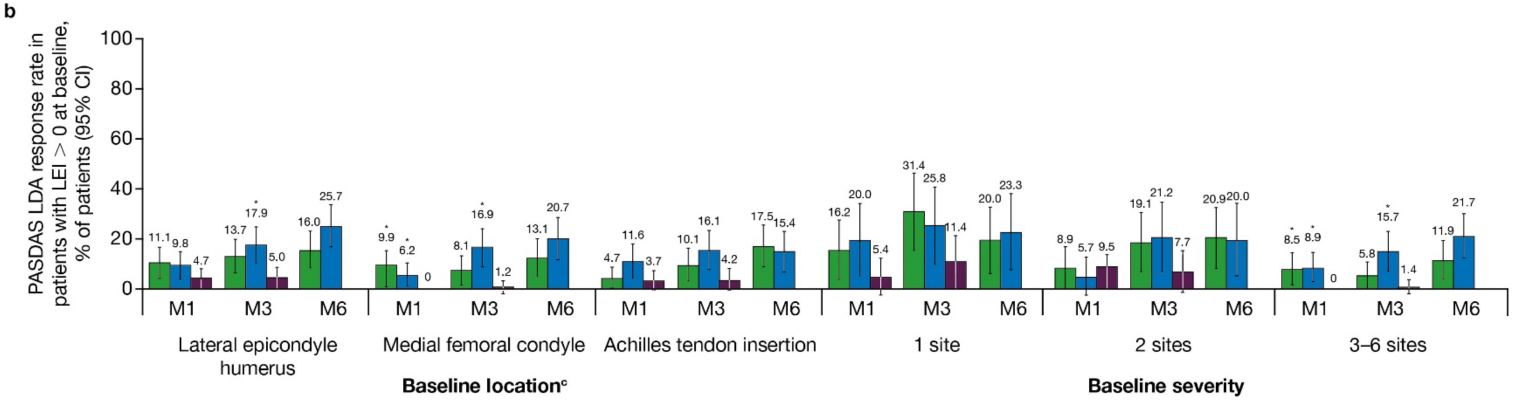

	M1	M3	M6	M1	M3	M6	M1	M3	M6
Tofacitinib 5 mg BID *n/N*:	11/99	13/95	15/94	9/91	7/86	11/84	4/85	8/79	14/80
Tofacitinib 10 mg BID *n/N*:	11/112	19/106	27/105	6/97	15/89	18/87	10/86	13/81	12/78
Placebo *n/N*:	5/106	5/100		0/88	1/83		3/81	3/72	

	M1	M3	M6	M1	M3	M6	M1	M3	M6
Tofacitinib 5 mg BID *n/N*:	6/37	11/35	7/35	4/45	8/24	9/43	6/71	4/69	8/67
Tofacitinib 10 mg BID *n/N*:	6/30	8/31	7/30	2/35	7/33	6/30	8/90	13/83	18/83
Placebo *n/N*:	2/37	4/35		4/42	3/39		0/75	1/70	

c

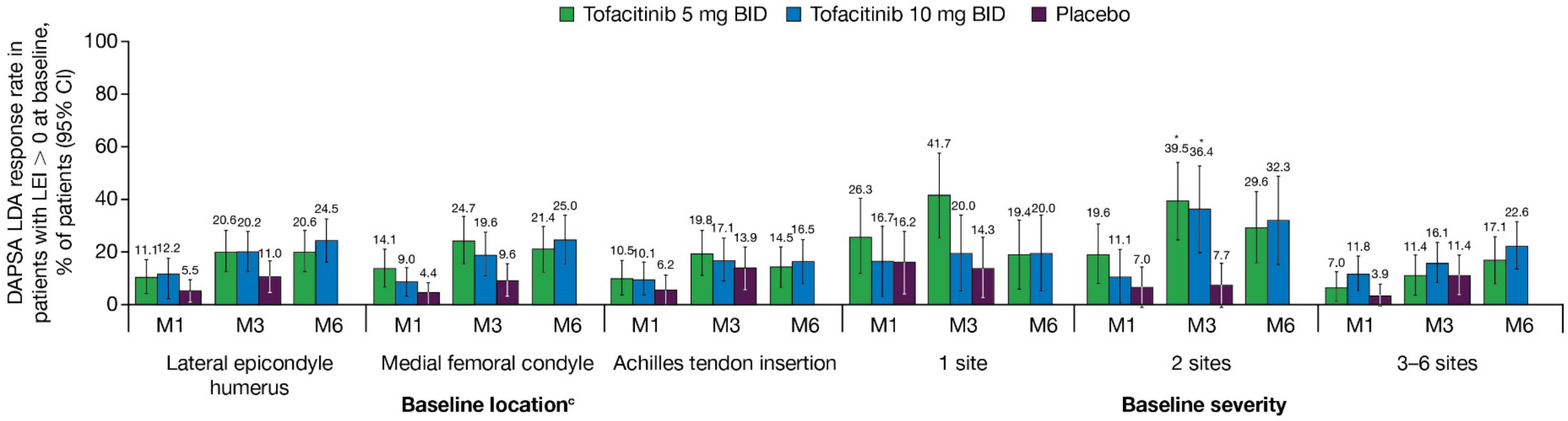

Tofacitinib 5 mg BID *n/N*:	11/99	20/97	20/97	13/92	22/89	19/89	9/86	16/81	12/83	10/38	15/36	7/36	9/46	17/43	13/44	5/71	8/70	12/70
Tofacitinib 10 mg BID *n/N*:	14/115	22/109	26/106	9/100	18/92	22/88	9/89	14/82	13/79	5/30	6/30	6/30	4/36	12/33	10/31	11/93	14/87	19/84
Placebo *n/N*:	6/109	11/100		4/90	8/83		5/81	10/72		6/37	5/35		3/43	3/39		3/77	8/70	

d

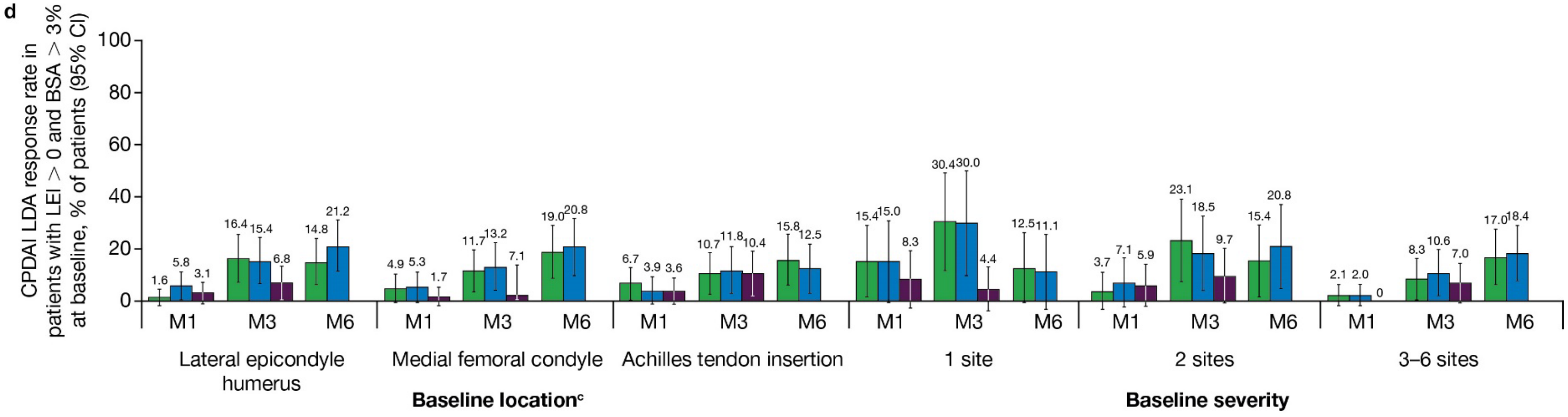

Tofacitinib 5 mg BID *n/N*:	1/63	10/61	9/61	3/61	7/60	11/58	4/60	6/56	9/57	4/26	7/23	3/24	1/27	6/26	4/26	1/48	4/48	8/47
Tofacitinib 10 mg BID *n/N*:	4/69	10/65	14/66	3/57	7/53	11/53	2/52	6/51	6/48	3/20	6/20	2/18	2/28	5/27	5/24	1/50	5/47	9/49
Placebo *n/N*:	2/65	4/59		1/58	4/56		2/55	5/48		2/24	1/23		2/34	3/31		0/47	3/43	

e

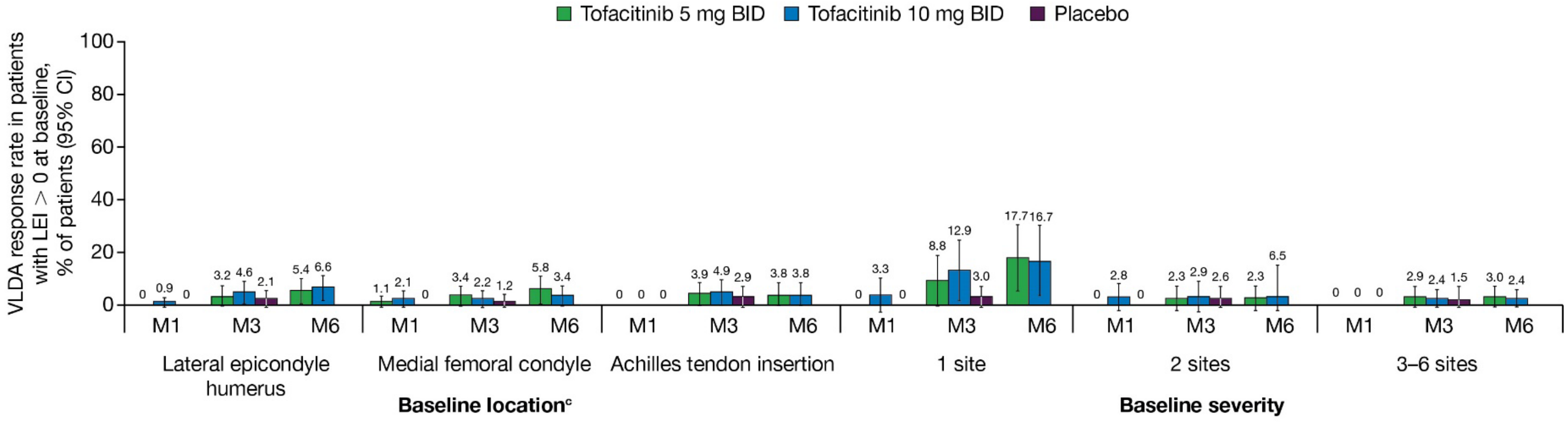

	Lateral epicondyle humerus M1	M3	M6	Medial femoral condyle M1	M3	M6	Achilles tendon insertion M1	M3	M6	1 site M1	M3	M6	2 sites M1	M3	M6	3–6 sites M1	M3	M6
Tofacitinib 5 mg BID *n/N*:	0/98	3/94	5/93	1/91	3/88	5/86	0/85	3/78	3/80	0/37	3/34	6/34	0/46	1/43	1/44	0/70	2/69	2/67
Tofacitinib 10 mg BID *n/N*:	1/112	5/108	7/106	2/96	2/91	3/88	0/85	4/81	3/80	1/30	4/31	5/30	1/36	1/34	2/31	0/89	2/85	2/84
Placebo *n/N*:	0/105	2/95		0/88	1/83		0/80	2/70		0/36	1/33		0/43	1/39		0/74	1/68	

f

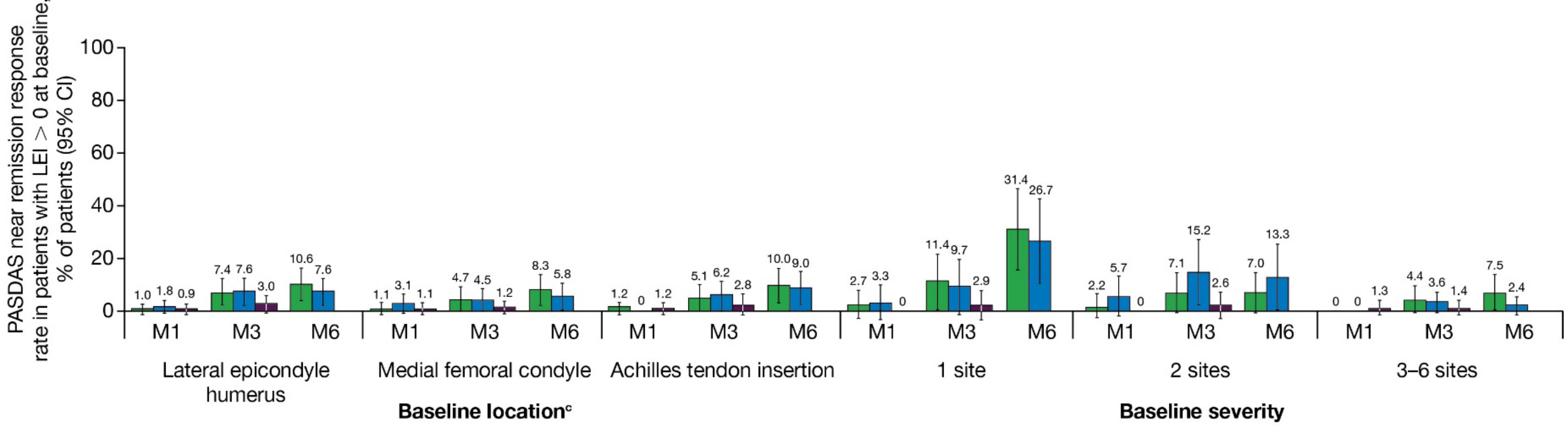

	Lateral epicondyle humerus M1	M3	M6	Medial femoral condyle M1	M3	M6	Achilles tendon insertion M1	M3	M6	1 site M1	M3	M6	2 sites M1	M3	M6	3–6 sites M1	M3	M6
Tofacitinib 5 mg BID *n/N*:	1/99	7/95	10/94	1/91	4/86	7/84	1/85	4/79	8/80	1/37	4/35	11/35	1/45	3/42	3/43	0/71	3/69	5/67
Tofacitinib 10 mg BID *n/N*:	2/112	8/106	8/105	3/97	4/89	5/87	0/86	5/81	7/78	1/30	3/31	8/30	2/35	5/33	4/30	0/90	3/83	2/83
Placebo *n/N*:	1/106	3/100		1/88	1/83		1/81	2/72		0/37	1/35		0/42	1/39		1/75	1/70	

g

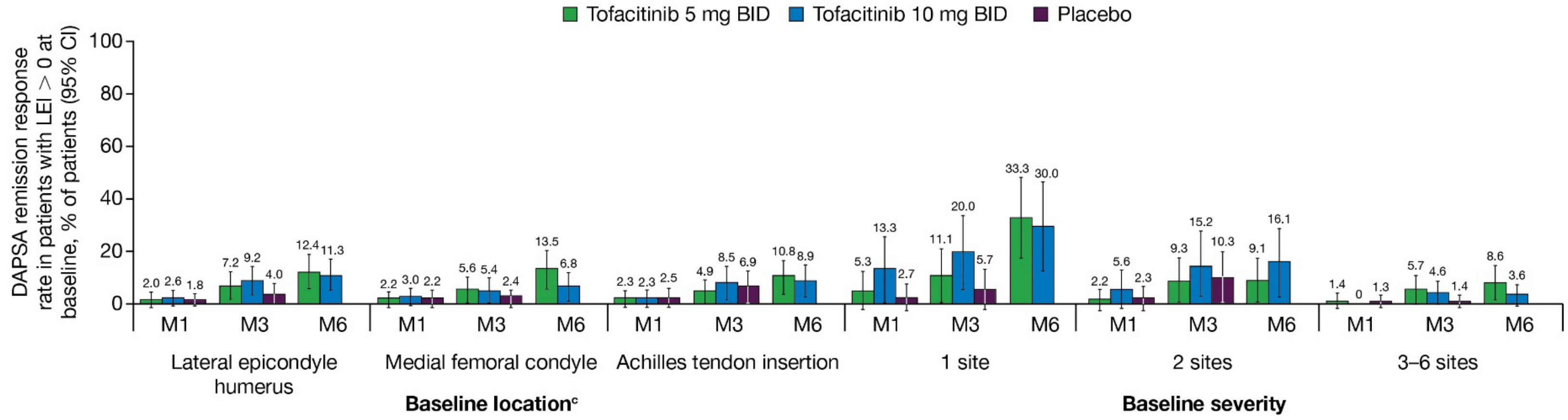

	M1	M3	M6	M1	M3	M6	M1	M3	M6	M1	M3	M6	M1	M3	M6	M1	M3	M6
Tofacitinib 5 mg BID *n/N*:	2/99	7/97	12/97	2/92	5/89	12/89	2/86	4/81	9/83	2/38	4/36	12/36	1/46	4/43	4/44	1/71	4/70	6/70
Tofacitinib 10 mg BID *n/N*:	3/115	10/109	12/106	3/100	5/92	6/88	2/89	7/82	7/79	4/30	6/30	9/30	2/36	5/33	5/31	0/93	4/87	3/84
Placebo *n/N*:	2/109	4/100		2/90	2/83		2/81	5/72		1/37	2/35		1/43	4/39		1/77	1/70	

h

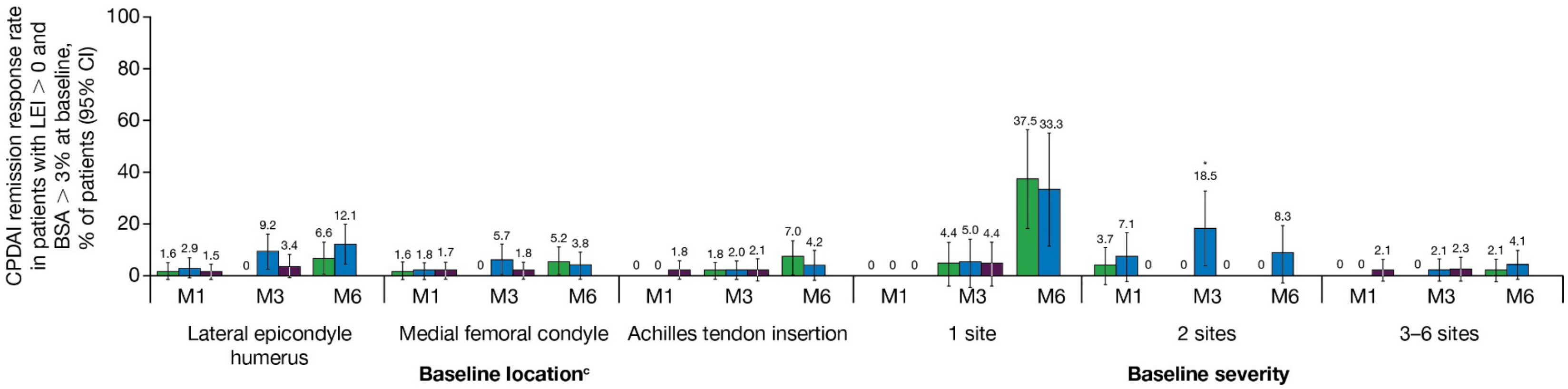

	M1	M3	M6	M1	M3	M6	M1	M3	M6	M1	M3	M6	M1	M3	M6	M1	M3	M6
Tofacitinib 5 mg BID *n/N*:	1/63	0/61	4/61	1/61	0/60	3/58	0/60	1/56	4/57	0/26	1/23	9/24	1/27	0/26	0/26	0/48	0/48	1/47
Tofacitinib 10 mg BID *n/N*:	2/69	6/65	8/66	1/57	3/53	2/53	0/52	1/51	2/48	0/20	1/20	6/18	2/28	5/27	2/24	0/50	1/47	2/49
Placebo *n/N*:	1/65	2/59		1/58	1/56		1/55	1/48		0/24	1/23		0/34	0/31		1/47	1/43	

LDA and remission, based on MDA (≥ 5/7 criteria; included patients who fulfilled 6/7 and 7/7 MDA criteria)/VLDA (7/7 MDA criteria), PASDAS (> 1.9–< 3.2/≤ 1.9 [near remission]), DAPSA (> 4–≤ 14/≤ 4), and CPDAI (> 2–≤ 4/≤ 2). *Indicates a comparison where the 95% CI for tofacitinib does not overlap with the 95% CI for placebo

[a]PASDAS LDA/near remission. [b]Assessed only in patients with LEI > 0 and BSA ≥ 3% at baseline. [c]Each site was assessed bilaterally, and results were combined. BID, twice daily; BSA, body surface area; CI, confidence interval; CPDAI, Composite Psoriatic Disease Activity in Psoriatic Arthritis; DAPSA, Disease Activity Index for Psoriatic Arthritis; LDA, low disease activity; LEI, Leeds Enthesitis Index; M, month; MDA, minimal disease activity; *N*, total number of patients with LEI > 0 at baseline in that particular location or number of affected sites; *n*, number of patients achieving outcome; PASDAS, Psoriatic Arthritis Disease Activity Score; VLDA, very low disease activity

Supplementary Fig. 2 LDA and remission rates, based on MDA or VLDA, PASDAS[a], DAPSA, and CPDAI[b] criteria (patients with SPARCC > 0 at baseline)

a

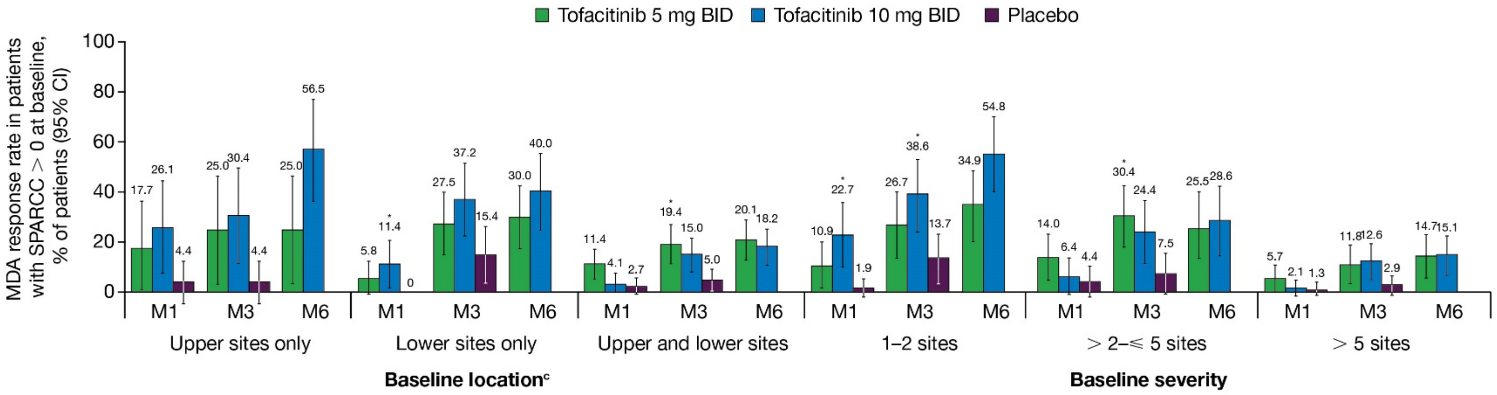

	M1	M3	M6	M1	M3	M6	M1	M3	M6	M1	M3	M6	M1	M3	M6	M1	M3	M6
Tofacitinib 5 mg BID *n/N*:	3/17	4/16	4/16	3/52	14/51	15/50	12/105	20/103	21/101	5/46	12/45	15/43	8/57	17/56	14/55	4/70	8/68	10/68
Tofacitinib 10 mg BID *n/N*:	6/23	7/23	13/23	5/44	16/43	16/40	5/121	17/113	20/110	10/44	17/44	23/42	3/47	11/45	12/42	2/94	11/87	13/86
Placebo *n/N*:	1/23	1/23		0/41	6/39		3/111	5/100		1/53	7/51		2/45	3/40		1/75	2/69	

b

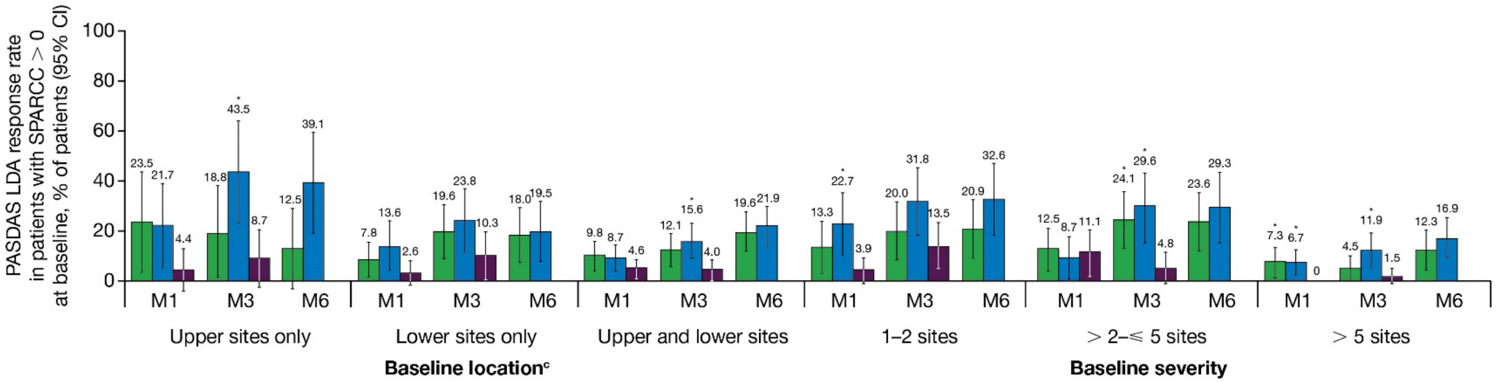

	M1	M3	M6	M1	M3	M6	M1	M3	M6	M1	M3	M6	M1	M3	M6	M1	M3	M6
Tofacitinib 5 mg BID *n/N*:	4/17	3/16	2/16	4/51	10/51	9/50	10/102	12/99	19/97	6/45	9/45	9/43	7/56	13/54	13/55	5/69	3/67	8/65
Tofacitinib 10 mg BID *n/N*:	5/23	10/23	9/23	6/44	10/42	8/41	10/115	17/109	23/105	10/44	14/44	14/43	4/46	13/44	12/41	6/90	10/84	14/83
Placebo *n/N*:	1/23	2/23		1/39	4/39		5/109	4/100		2/52	7/52		5/45	2/42		0/74	1/68	

c

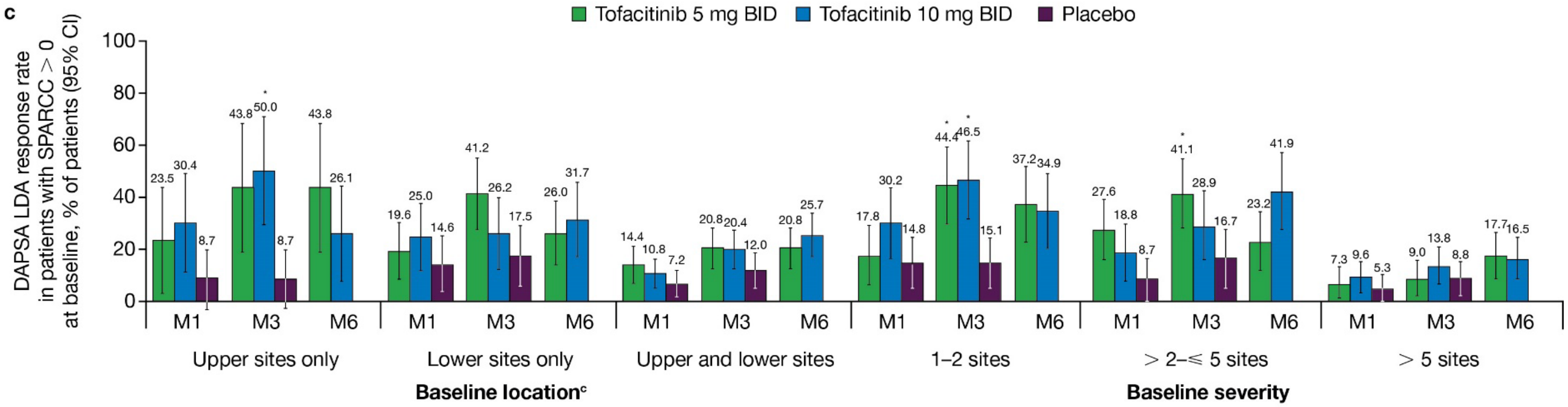

	Upper sites only M1	M3	M6	Lower sites only M1	M3	M6	Upper and lower sites M1	M3	M6	1–2 sites M1	M3	M6	> 2–≤ 5 sites M1	M3	M6	> 5 sites M1	M3	M6
Tofacitinib 5 mg BID *n/N*:	4/17	7/16	7/16	10/51	21/51	13/50	15/104	21/101	21/101	8/45	20/45	16/43	16/58	23/56	13/56	5/69	6/67	12/68
Tofacitinib 10 mg BID *n/N*:	7/23	11/22	6/23	11/44	11/42	13/41	13/120	23/113	28/109	13/43	20/43	15/43	9/48	13/45	18/43	9/94	12/87	14/85
Placebo *n/N*:	2/23	2/23		6/41	7/40		8/111	12/100		8/54	8/53		4/46	7/42		4/75	6/68	

d

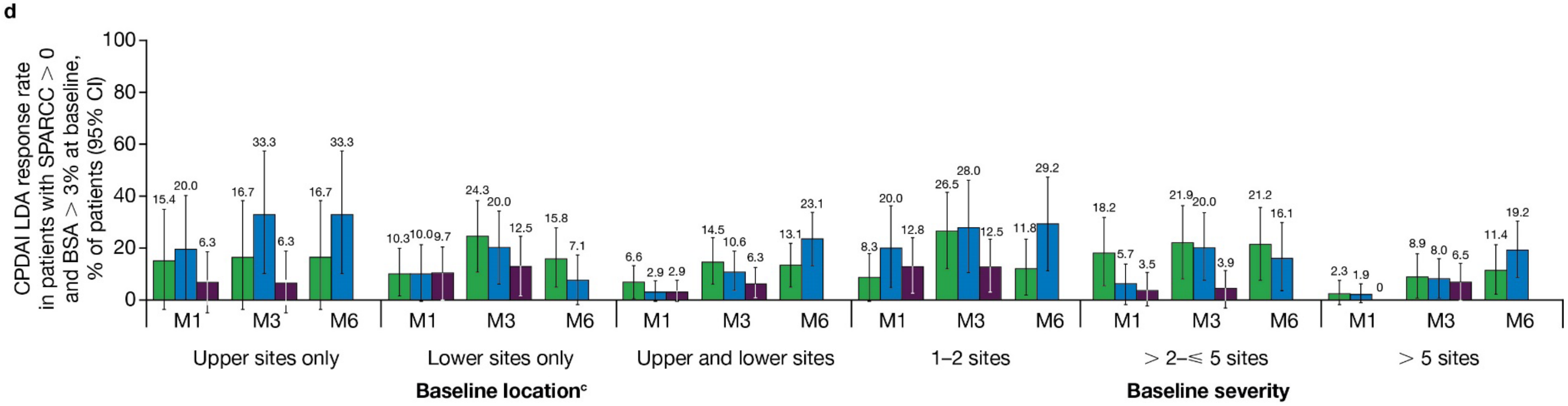

	Upper sites only M1	M3	M6	Lower sites only M1	M3	M6	Upper and lower sites M1	M3	M6	1–2 sites M1	M3	M6	> 2–≤ 5 sites M1	M3	M6	> 5 sites M1	M3	M6
Tofacitinib 5 mg BID *n/N*:	2/13	2/12	2/12	4/39	9/37	6/38	4/61	9/62	8/61	3/36	9/34	4/34	6/33	7/32	7/33	1/44	4/45	5/44
Tofacitinib 10 mg BID *n/N*:	3/15	5/15	5/15	3/30	6/30	2/28	2/70	7/66	15/65	5/25	7/25	7/24	2/35	7/35	5/31	1/54	4/50	10/52
Placebo *n/N*:	1/16	1/16		3/31	4/32		2/70	4/64		5/39	5/40		1/29	1/26		0/49	3/46	

e

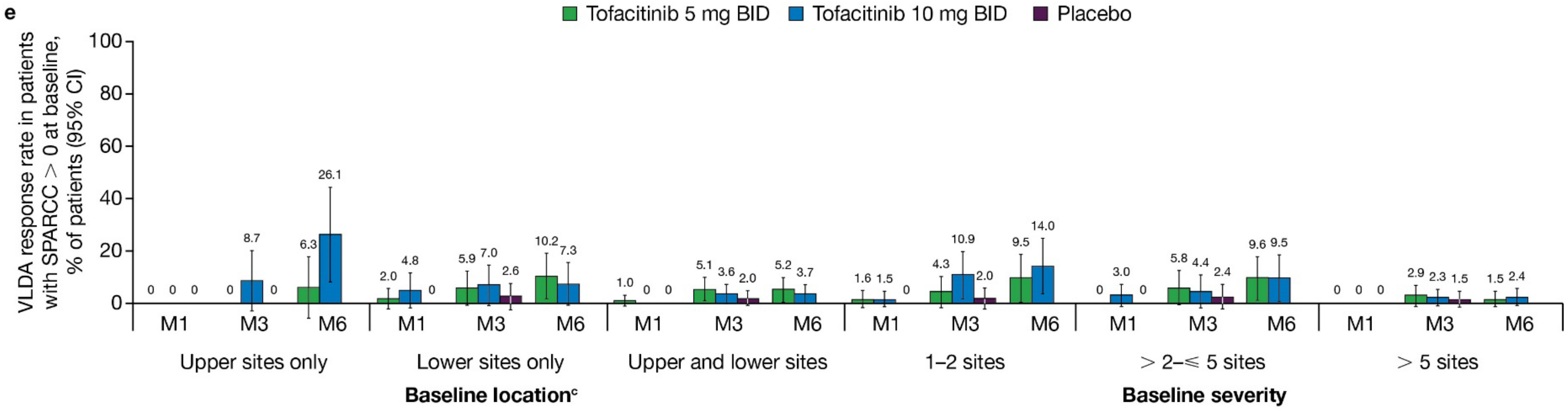

	Upper sites only M1	Upper sites only M3	Upper sites only M6	Lower sites only M1	Lower sites only M3	Lower sites only M6	Upper and lower sites M1	Upper and lower sites M3	Upper and lower sites M6	1–2 sites M1	1–2 sites M3	1–2 sites M6	> 2–≤ 5 sites M1	> 2–≤ 5 sites M3	> 2–≤ 5 sites M6	> 5 sites M1	> 5 sites M3	> 5 sites M6
Tofacitinib 5 mg BID *n/N*:	0/17	0/16	1/16	1/50	3/51	5/49	1/103	5/98	5/96	1/63	2/47	4/42	0/87	3/52	5/52	0/95	2/68	1/66
Tofacitinib 10 mg BID *n/N*:	0/23	2/23	6/23	2/42	3/43	3/41	0/117	4/112	4/109	1/66	5/46	6/43	2/67	2/45	4/42	0/123	2/87	2/85
Placebo *n/N*:	0/23	0/22		0/40	1/39		0/109	2/98		0/78	1/50		0/59	1/41		0/109	1/67	

f

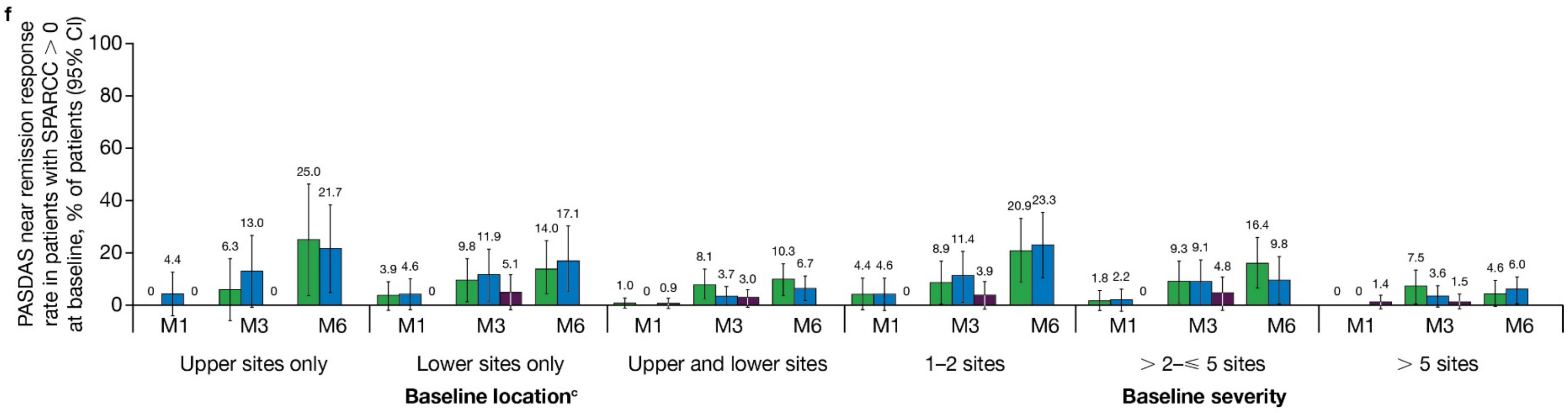

	Upper sites only M1	Upper sites only M3	Upper sites only M6	Lower sites only M1	Lower sites only M3	Lower sites only M6	Upper and lower sites M1	Upper and lower sites M3	Upper and lower sites M6	1–2 sites M1	1–2 sites M3	1–2 sites M6	> 2–≤ 5 sites M1	> 2–≤ 5 sites M3	> 2–≤ 5 sites M6	> 5 sites M1	> 5 sites M3	> 5 sites M6
Tofacitinib 5 mg BID *n/N*:	0/17	1/16	4/16	2/51	5/51	7/50	1/102	8/99	10/97	2/45	4/45	9/43	1/56	5/54	9/55	0/69	5/67	3/65
Tofacitinib 10 mg BID *n/N*:	1/23	3/23	5/23	2/44	5/42	7/41	0/115	4/109	7/105	2/44	5/44	10/43	1/46	4/44	4/41	0/90	3/84	5/83
Placebo *n/N*:	0/23	0/23		0/39	2/39		1/109	3/100		0/52	2/52		0/45	2/42		1/74	1/68	

g

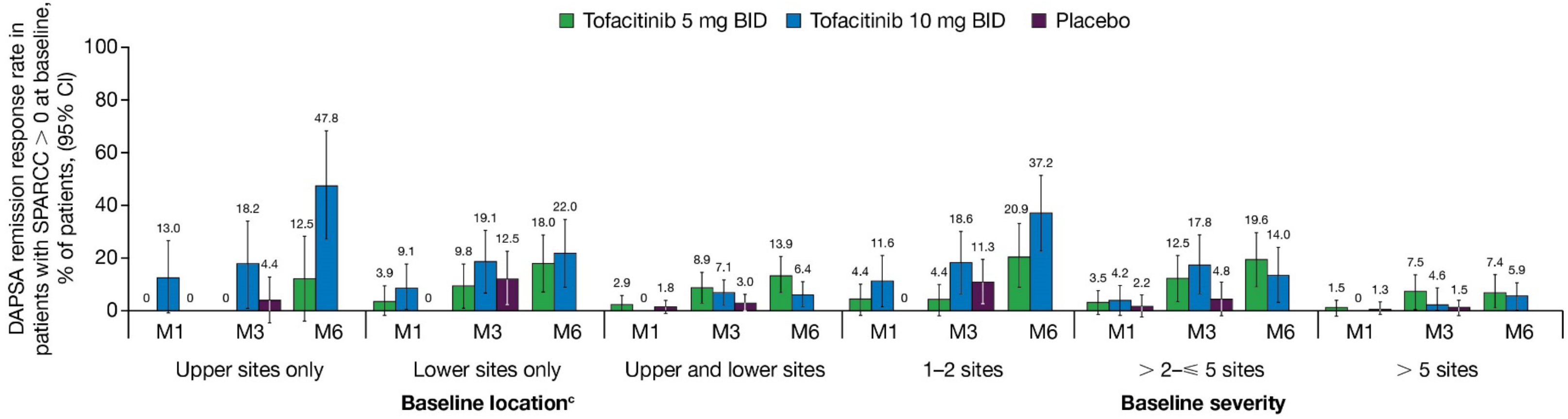

Tofacitinib 5 mg BID *n/N*:	0/17	0/16	2/16	2/51	5/51	9/50	3/104	9/101	14/101	2/45	2/45	9/43	2/58	7/56	11/56	1/69	5/67	5/68
Tofacitinib 10 mg BID *n/N*:	3/23	4/22	11/23	4/44	8/42	9/41	0/120	8/113	7/109	5/43	8/43	16/43	2/48	8/45	6/43	0/94	4/87	5/85
Placebo *n/N*:	0/23	1/23		0/41	5/40		2/111	3/100		0/54	6/53		1/46	2/42		1/75	1/68	

h

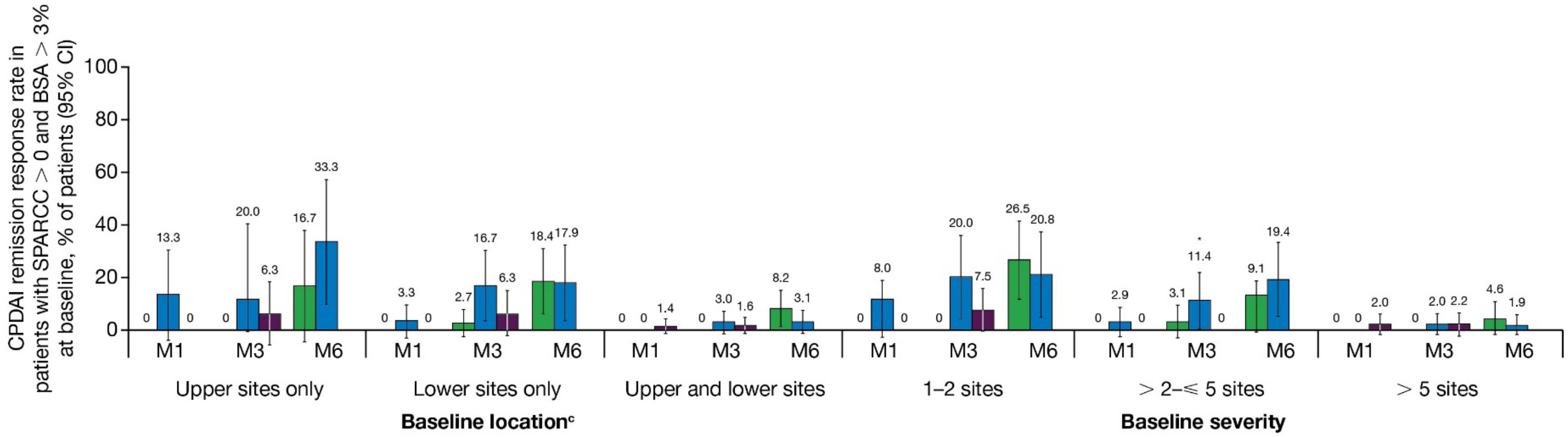

Tofacitinib 5 mg BID *n/N*:	0/13	0/12	2/12	0/39	1/37	7/38	0/61	0/62	5/61	0/36	0/34	9/34	0/33	1/32	3/33	0/44	0/45	2/44
Tofacitinib 10 mg BID *n/N*:	2/15	3/15	5/15	1/30	5/30	5/28	0/70	2/66	2/65	2/25	5/25	5/24	1/35	4/35	6/31	0/54	1/50	1/52
Placebo *n/N*:	0/16	1/16		0/31	2/32		1/70	1/64		0/39	3/40		0/29	0/26		1/49	1/46	

LDA and remission, based on MDA ($\geq$ 5/7 criteria; included patients who fulfilled 6/7 and 7/7 MDA criteria)/VLDA (7/7 MDA criteria), PASDAS (> 1.9–< 3.2/$\leq$ 1.9 [near remission]), DAPSA (> 4–$\leq$ 14/$\leq$ 4), and CPDAI (> 2–$\leq$ 4/$\leq$ 2). *Indicates a comparison where the 95% CI for tofacitinib does not overlap with the 95% CI for placebo. [a]PASDAS LDA/near remission. [b]Assessed only in patients with SPARCC > 0 and BSA $\geq$ 3% at baseline. [c]Each site was assessed bilaterally, and results were combined. BID, twice daily; BSA, body surface area; CI, confidence interval; CPDAI, Composite Psoriatic Disease Activity in Psoriatic Arthritis; DAPSA, Disease Activity Index for Psoriatic Arthritis; LDA, low disease activity; M, month; MDA, minimal disease activity; *N*, total number of patients with SPARCC > 0 at baseline in that particular location or number of affected sites; *n*, number of patients achieving outcome; PASDAS, Psoriatic Arthritis Disease Activity Score; SPARCC, Spondyloarthritis Research Consortium of Canada Enthesitis Index; VLDA, very low disease activity

Supplementary Fig. 3 LDA and remission rates, based on MDA or VLDA, PASDAS[a], DAPSA, and CPDAI[b] criteria (patients with LEI = 0 and SPARCC = 0 at baseline)

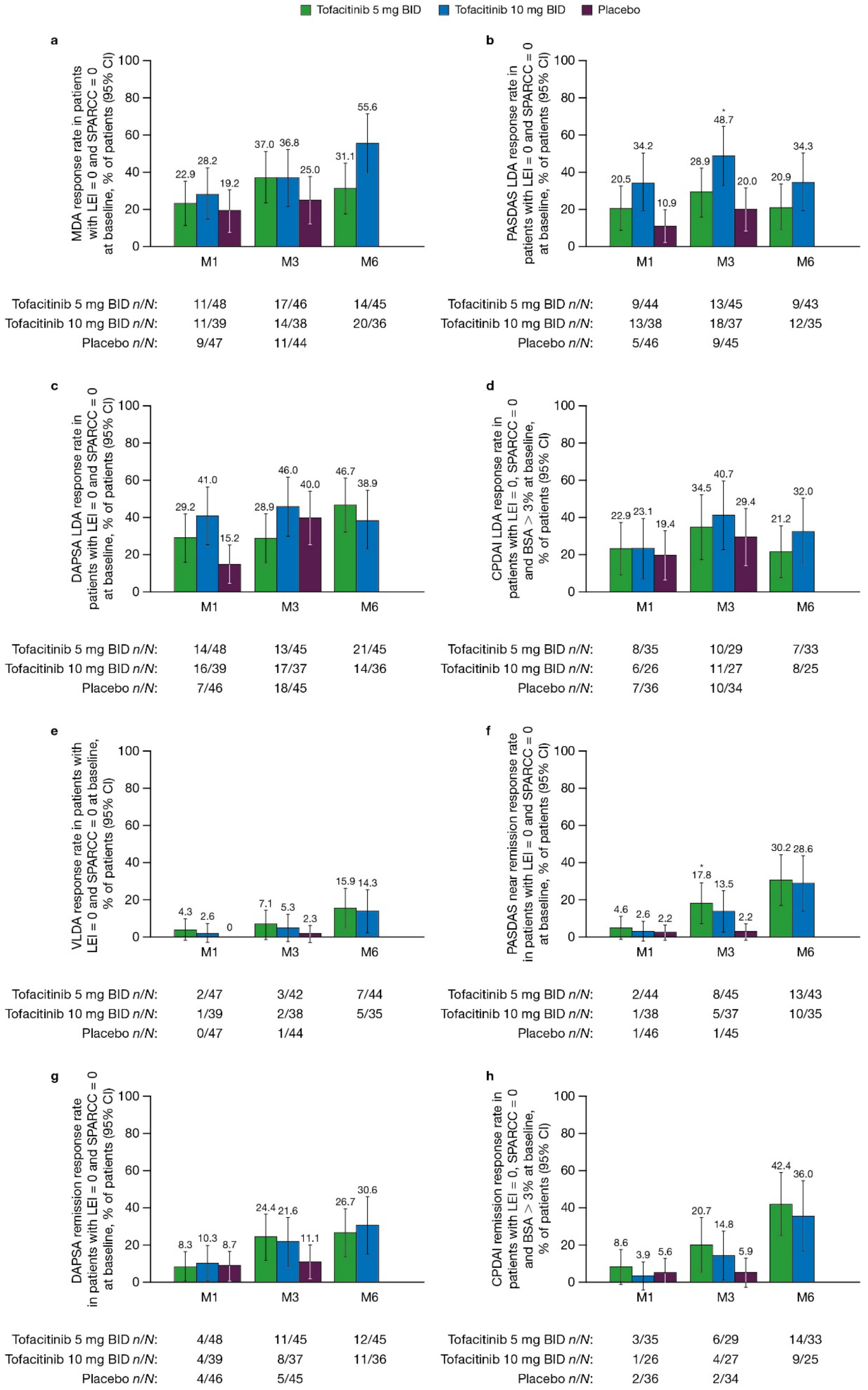

LDA and remission, based on MDA (≥ 5/7 criteria; included patients who fulfilled 6/7 and 7/7 MDA criteria)/VLDA (7/7 MDA criteria), PASDAS (> 1.9–< 3.2/≤ 1.9 [near remission]), DAPSA (> 4–≤ 14/≤ 4), and CPDAI (> 2–≤ 4/≤ 2). *Indicates a comparison where the 95% CI for tofacitinib does not overlap with the 95% CI for placebo. [a]PASDAS LDA/near remission. [b]Assessed only in patients with LEI = 0 and SPARCC = 0 and BSA ≥ 3% at baseline. BID, twice daily; BSA, body surface area; CI, confidence interval; CPDAI, Composite Psoriatic Disease Activity in Psoriatic Arthritis; DAPSA, Disease Activity Index for Psoriatic Arthritis; LDA, low disease activity; LEI, Leeds Enthesitis Index; M, month; MDA, minimal disease activity; *N*, total number of patients with LEI = 0 and SPARCC = 0 at baseline; *n*, number of patients achieving outcome; PASDAS, Psoriatic Arthritis Disease Activity Score; SPARCC, Spondyloarthritis Research Consortium of Canada Enthesitis Index; VLDA, very low disease activity

Supplementary Fig. 4 FACIT-F total and arthritis pain (VAS) scores (patients with LEI > 0/SPARCC > 0/LEI = 0 and SPARCC = 0 at baseline)

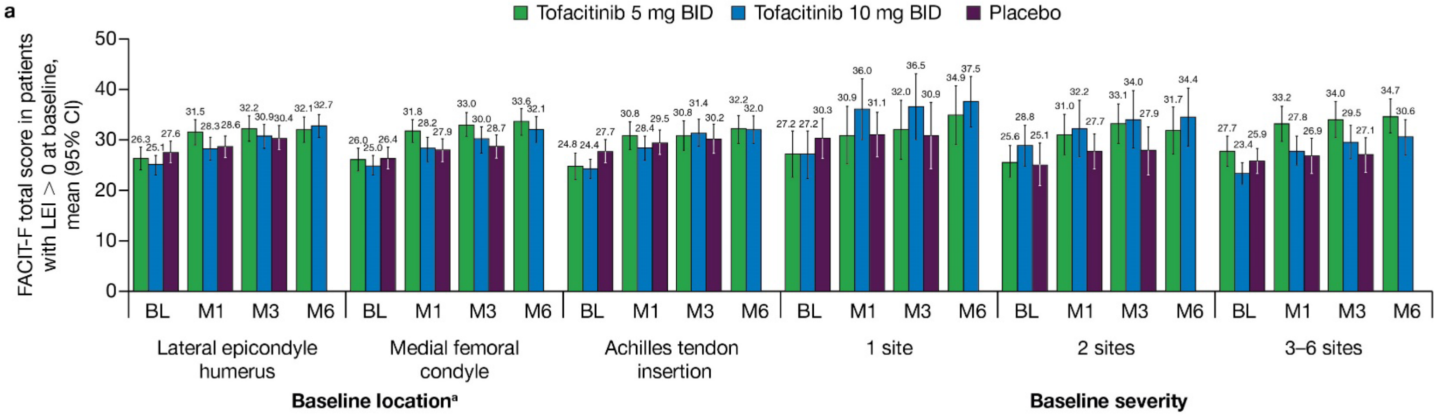

	Lat. epicondyle BL	M1	M3	M6	Med. femoral BL	M1	M3	M6	Achilles BL	M1	M3	M6	1 site BL	M1	M3	M6	2 sites BL	M1	M3	M6	3–6 sites BL	M1	M3	M6
Tofacitinib 5 mg BID *N*:	99	95	93	92	92	90	87	86	83	82	77	79	27	19	19	19	38	24	22	23	54	45	45	44
Tofacitinib 10 mg BID *N*:	117	114	106	105	99	98	89	87	90	88	80	78	22	15	15	15	29	21	21	19	66	51	46	46
Placebo *N*:	107	104	96		89	86	81		78	77	68		24	17	16		29	21	18		58	44	41	

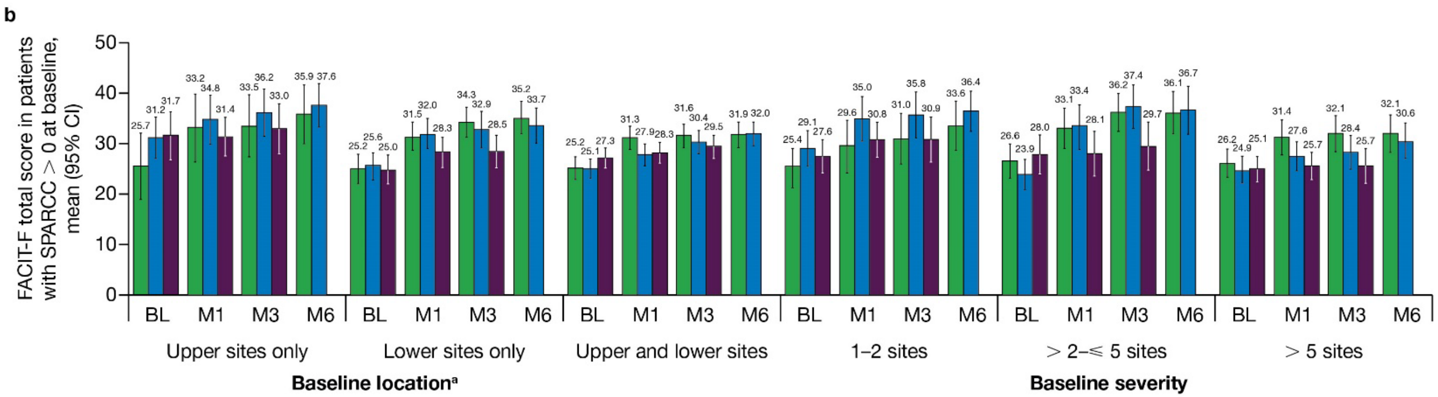

	Upper only BL	M1	M3	M6	Lower only BL	M1	M3	M6	Upper and lower BL	M1	M3	M6	1–2 sites BL	M1	M3	M6	> 2–≤ 5 sites BL	M1	M3	M6	> 5 sites BL	M1	M3	M6
Tofacitinib 5 mg BID *N*:	18	17	16	16	53	52	51	50	104	104	101	100	35	23	24	22	45	32	30	30	54	44	44	44
Tofacitinib 10 mg BID *N*:	23	23	23	23	45	44	43	41	122	119	110	108	35	24	24	23	36	27	27	25	66	52	47	47
Placebo *N*:	23	22	22		41	41	40		113	110	101		39	25	25		30	22	20		56	43	40	

c

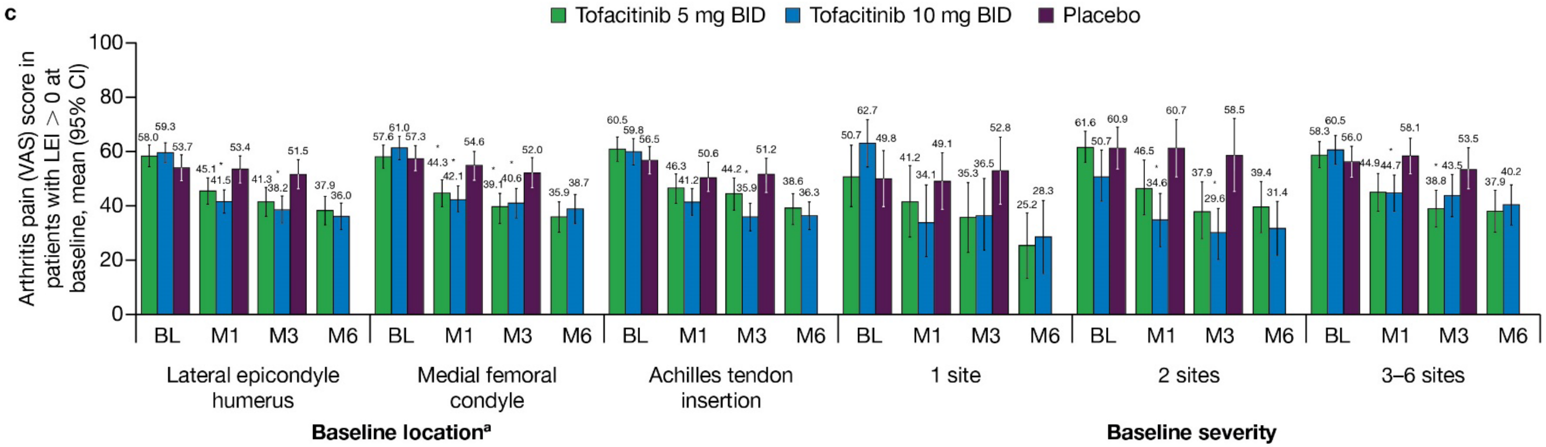

	Lateral epicondyle humerus BL	M1	M3	M6	Medial femoral condyle BL	M1	M3	M6	Achilles tendon insertion BL	M1	M3	M6	1 site BL	M1	M3	M6	2 sites BL	M1	M3	M6	3–6 sites BL	M1	M3	M6
Tofacitinib 5 mg BID *N*:	101	100	98	97	93	93	91	89	86	86	82	83	27	19	19	19	38	24	23	23	54	45	45	44
Tofacitinib 10 mg BID *N*:	119	116	109	107	100	100	92	89	92	90	83	80	22	15	15	15	29	21	21	19	66	51	47	46
Placebo *N*:	110	109	100		91	90	84		81	81	72		24	17	16		29	21	18		58	45	41	

d

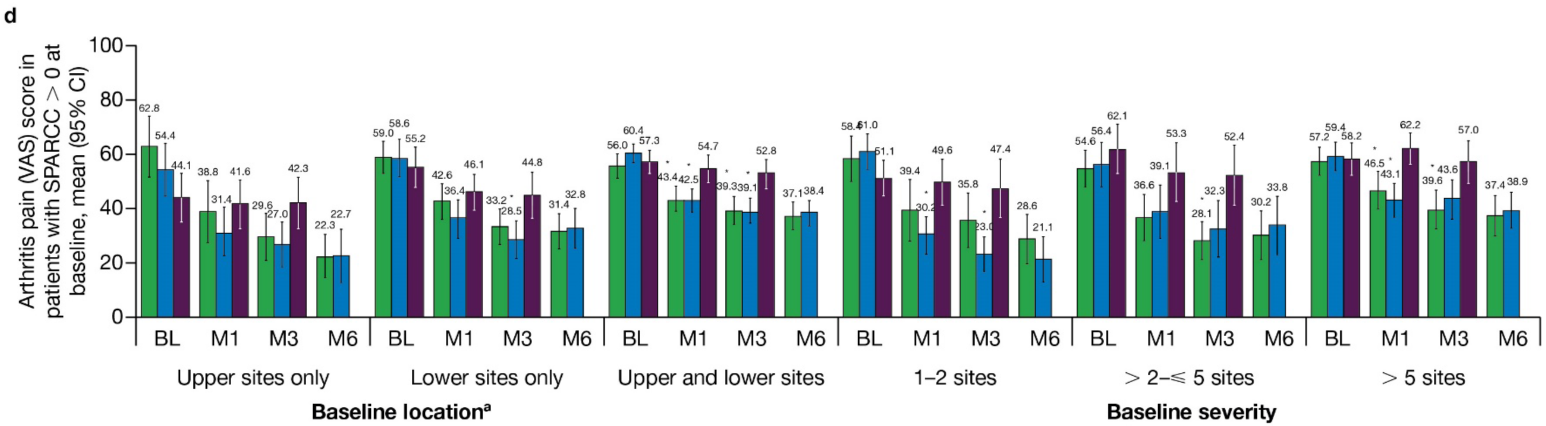

	Upper sites only BL	M1	M3	M6	Lower sites only BL	M1	M3	M6	Upper and lower sites BL	M1	M3	M6	1–2 sites BL	M1	M3	M6	> 2–≤ 5 sites BL	M1	M3	M6	> 5 sites BL	M1	M3	M6
Tofacitinib 5 mg BID *N*:	18	17	16	16	53	52	51	50	105	105	103	101	35	23	24	22	45	32	31	30	54	44	44	44
Tofacitinib 10 mg BID *N*:	23	23	22	23	45	44	43	41	123	121	113	110	35	24	24	23	36	27	27	25	66	52	48	47
Placebo *N*:	23	23	23		41	41	40		113	111	101		39	25	25		30	23	20		56	43	40	

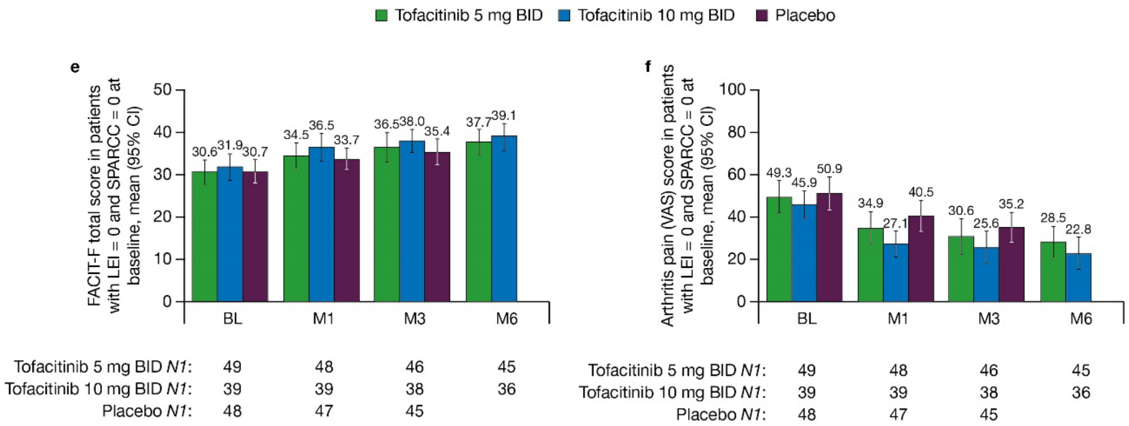

*Indicates a comparison where the 95% CI for tofacitinib does not overlap with the 95% CI for placebo. [a]Each site was assessed bilaterally, and results were combined. BID, twice daily; BL, baseline; CI, confidence interval; FACIT-F, Functional Assessment of Chronic Illness Therapy-Fatigue; LEI, Leeds Enthesitis Index; M, month; N, total number of patients with LEI > 0 or SPARCC > 0 at baseline in that particular location or number of affected sites; *N1*, total number of patients with LEI = 0 and SPARCC = 0 at baseline; SPARCC, Spondyloarthritis Research Consortium of Canada Enthesitis Index; VAS, Visual Analog Score

References

1. Coates LC, Fransen J, Helliwell PS. Defining minimal disease activity in psoriatic arthritis: a proposed objective target for treatment. Ann Rheum Dis. 2010;69:48-53.
2. Coates LC, Helliwell PS. Defining low disease activity states in psoriatic arthritis using novel composite disease instruments. J Rheumatol. 2016;43:371-5.
3. Helliwell PS, FitzGerald O, Fransen J, Gladman DD, Kreuger GG, Callis-Duffin K, et al. The development of candidate composite disease activity and responder indices for psoriatic arthritis (GRACE project). Ann Rheum Dis. 2013;72:986-91.
4. Perruccio AV, Got M, Li S, Ye Y, Gladman DD, Chandran V. Treating psoriatic arthritis to target: defining the psoriatic arthritis disease activity score that reflects a state of minimal disease activity. J Rheumatol. 2020;47:362-8.
5. Schoels M. Psoriatic arthritis indices. Clin Exp Rheumatol. 2014;32:S109-S12.
6. Schoels MM, Aletaha D, Alasti F, Smolen JS. Disease activity in psoriatic arthritis (PsA): defining remission and treatment success using the DAPSA score. Ann Rheum Dis. 2016;75:811-8.
7. Mumtaz A, Gallagher P, Kirby B, Waxman R, Coates LC, Veale JD, et al. Development of a preliminary composite disease activity index in psoriatic arthritis. Ann Rheum Dis. 2011;70:272-7.
8. Helliwell PS, FitzGerald O, Fransen J. Composite disease activity and responder indices for psoriatic arthritis: a report from the GRAPPA 2013 meeting on development of cutoffs for both disease activity states and response. J Rheumatol. 2014;41:1212-7.
